# Cell-Penetrating Peptides Selectively Cross the Blood-Brain Barrier *In Vivo*

**DOI:** 10.1371/journal.pone.0139652

**Published:** 2015-10-14

**Authors:** Sofie Stalmans, Nathalie Bracke, Evelien Wynendaele, Bert Gevaert, Kathelijne Peremans, Christian Burvenich, Ingeborgh Polis, Bart De Spiegeleer

**Affiliations:** 1 Drug Quality and Registration (DruQuaR) Group, Faculty of Pharmaceutical Sciences, Ghent University, Ghent, Belgium; 2 Department of Veterinary Medical Imaging and Small Animal Orthopaedics, Faculty of Veterinary Medicine, Ghent University, Merelbeke, Belgium; 3 Department of Comparative Physiology and Biometrics, Faculty of Veterinary Medicine, Ghent University, Merelbeke, Belgium; 4 Department of Medicine and Clinical Biology of Small Animals, Faculty of Veterinary Medicine, Ghent University, Merelbeke, Belgium; Hanyang University, REPUBLIC OF KOREA

## Abstract

Cell-penetrating peptides (CPPs) are a group of peptides, which have the ability to cross cell membrane bilayers. CPPs themselves can exert biological activity and can be formed endogenously. Fragmentary studies demonstrate their ability to enhance transport of different cargoes across the blood-brain barrier (BBB). However, comparative, quantitative data on the BBB permeability of different CPPs are currently lacking. Therefore, the *in vivo* BBB transport characteristics of five chemically diverse CPPs, *i*.*e*. pVEC, SynB3, Tat 47–57, transportan 10 (TP10) and TP10-2, were determined. The results of the multiple time regression (MTR) analysis revealed that CPPs show divergent BBB influx properties: Tat 47–57, SynB3, and especially pVEC showed very high unidirectional influx rates of 4.73 μl/(g × min), 5.63 μl/(g × min) and 6.02 μl/(g × min), respectively, while the transportan analogs showed a negligible to low brain influx. Using capillary depletion, it was found that 80% of the influxed peptides effectively reached the brain parenchyma. Except for pVEC, all peptides showed a significant efflux out of the brain. Co-injection of pVEC with radioiodinated bovine serum albumin (BSA) did not enhance the brain influx of radiodionated BSA, indicating that pVEC does not itself significantly alter the BBB properties. A saturable mechanism could not be demonstrated by co-injecting an excess dose of non-radiolabeled CPP. No significant regional differences in brain influx were observed, with the exception for pVEC, for which the regional variations were only marginal. The observed BBB influx transport properties cannot be correlated with their cell-penetrating ability, and therefore, good CPP properties do not imply efficient brain influx.

## Introduction

Cell-penetrating peptides are a particular group of peptides that have the ability to cross cell membranes without causing a significant lethal membrane damage [1,2]. The exact mechanism of cellular entry remains controversial, but a consensus exists that endocytosis and a direct penetration mechanism are involved [3,4]. Drawing conclusions on the cellular uptake mechanism of CPPs is impeded by different factors. Firstly, a variety of techniques and experimental protocols are used to investigate these peptides [1]. Furthermore, multiple mechanisms can simultaneously be active and the employed mechanisms depend not only on the peptide studied but also on the cell type used, the peptide concentration and the attached cargo or label [3,4].

Since their discovery 20 years ago, hundreds of CPPs have already been described and can roughly be classified into three chemical groups: the cationic, amphipathic and hydrophobic CPPs [5]. The cationic CPPs contain a stretch of positive charges derived from arginine and lysine residues in their sequence, while the amphipathic peptides are characterized by a hydrophilic and hydrophobic part, mostly by adopting a helix structure. The hydrophobic CPPs are rich in apolar amino acids and have a low net charge. However, a clear overlap exists between these chemical groups, emphasizing that CPPs represent a chemically diverse group of peptides [1].

The majority of the CPPs are derived from naturally occurring proteins and peptides like heparin-binding proteins, DNA and/or RNA-binding proteins, homeoproteins, signal peptides, antimicrobial peptides and viral proteins [5,6]. For some CPPs, their cell-penetrating properties are linked to the function of the parent peptide or protein, but for other peptides, the function of the CPP sequence in the full-length parent protein is still unclear [5]. Traditionally, CPPs are considered to be inert molecules, but actually these peptides can exert a biological activity themselves [7,8]. Moreover, for some CPPs the biological function of the parent peptide or protein is conserved in the cell-penetrating sequence, a feature that can be therapeutically exploited [9–18]. However, up till now, only a limited number of studies have described the biological activity of CPPs although this information is indicative for potential side effects and relevant for future clinical applications [7,8]. The knowledge on the bioactivity of CPPs is particularly interesting for peptides having an endogenous [9–13,15–17,19–23] and viral or bacterial origin [14,24–32], as these CPPs can also be endogenously produced during metabolization of their parent protein or peptide.

As CPPs are able to cross cellular membranes, the question arises whether this means that they can also pass the blood-brain barrier (BBB), which protects the brain. The barrier function of the BBB is established by physical, transport as well as metabolic means, explaining its selective permeability for ions and (macro)molecules [33]. Some fragmentary studies are describing the ability of CPPs to reach the brain parenchyma both *in vivo* and *in vitro* [34]. However, almost all studies investigate the brain delivery of a CPP attached to a cargo, which is known to influence the cell-penetrating, as well as the BBB transport properties. Moreover, different techniques are hereby used: measuring the pharmacological effect of the attached cargo or following the fluorescently labeled construct using *in vivo* imaging or fluorescence microscopy techniques. A detailed overview of the currently available brain influx studies of CPPs can be found in the Supporting Information (S1 Table). Comparable, quantitative data on the BBB transport of CPPs are still lacking. Moreover, there is only limited knowledge on the BBB transport mechanism of CPPs. Only for the SynB vectors, which are cationic CPPs, an adsorptive-mediated translocation mechanism is proposed [35,36].

To evaluate whether different CPPs cross the BBB to the same extent, we quantitatively investigated the BBB transport of five chemically diverse CPPs with different cell-penetrating ability (Table 1): pVEC, SynB3, Tat 47–57, transportan 10 (TP10) and TP10-2. The peptides were selected based on the different groups of CPPs determined during the previously performed exploration of the chemical space [1]. In this study, their BBB influx transport rate, the parenchyma/capillary and the intra-brain regional distribution after brain uptake, as well as the efflux properties were investigated using an *in vivo* mouse model. The influence of pVEC on the BBB permeability was verified. Finally, the saturability of the brain influx mechanism of pVEC, TP10 and SynB3 was evaluated. Our results indicate that CPPs selectively pass the BBB as not all CPPs cross the BBB to the same extent.

**Table 1:** Overview of the characteristics of the CPPs selected for the investigation of their BBB transport properties.

Property	pVEC	TP10	TP10-2	SynB3	Tat 47–57
**Sequence**	LLIILRRRIRKQAHAHSK-NH_2_	AGYLLGKINLKALAALAKKIL-NH_2_	AGYLLGKINLKPLAALAKKIL-NH_2_	RRLSYSRRRF-NH_2_	YGRKKRRQRRR-NH_2_
**Molecular weight (Da)** ^1^	2208.8	2181.8	2207.8	1395.7	1558.9
**CPP chemical class** **²**	Amphipathic-cationic	Amphipathic-cationic	Amphipathic-cationic	Cationic	Cationic
**Cell uptake mechanism** ^2^ ^**,**^ ^3^	Direct penetration and/or endocytosis	Direct penetration and/or endocytosis	Direct penetration	Endocytosis	Direct penetration and/or endocytosis
**CP-response** **²**	1.318	1.641	0.749	0.126	0.309
**Log P** ^4^	-7.2	-3.7	-3.5	-4.7	-9.2
**pI** ^5^	11.6	10.4	10.4	11.5	11.7
**% charged residues** ^6^	44%	19%	19%	50%	73%

^1^Obtained from the certificate of analysis provided by the supplier.

^2^As described by Stalmans *et al*.[1].

^3^Uptake mechanisms already reported in literature.

^4^Log P calculated using Hyperchem 8.0 (Hypercube, Gainesville, FL, USA).

^5^Iso-electric point as calculated using MarvinSketch 5.10.3 (ChemAxon, Budapest, Hungary).

^6^Calculated as the ratio of the number of basic amino acids (R,K or H) and the total number of amino acid residues multiplied by 100.

## Results

### Blood-to-brain transport kinetics of cell-penetrating peptides

**Fig 1:**
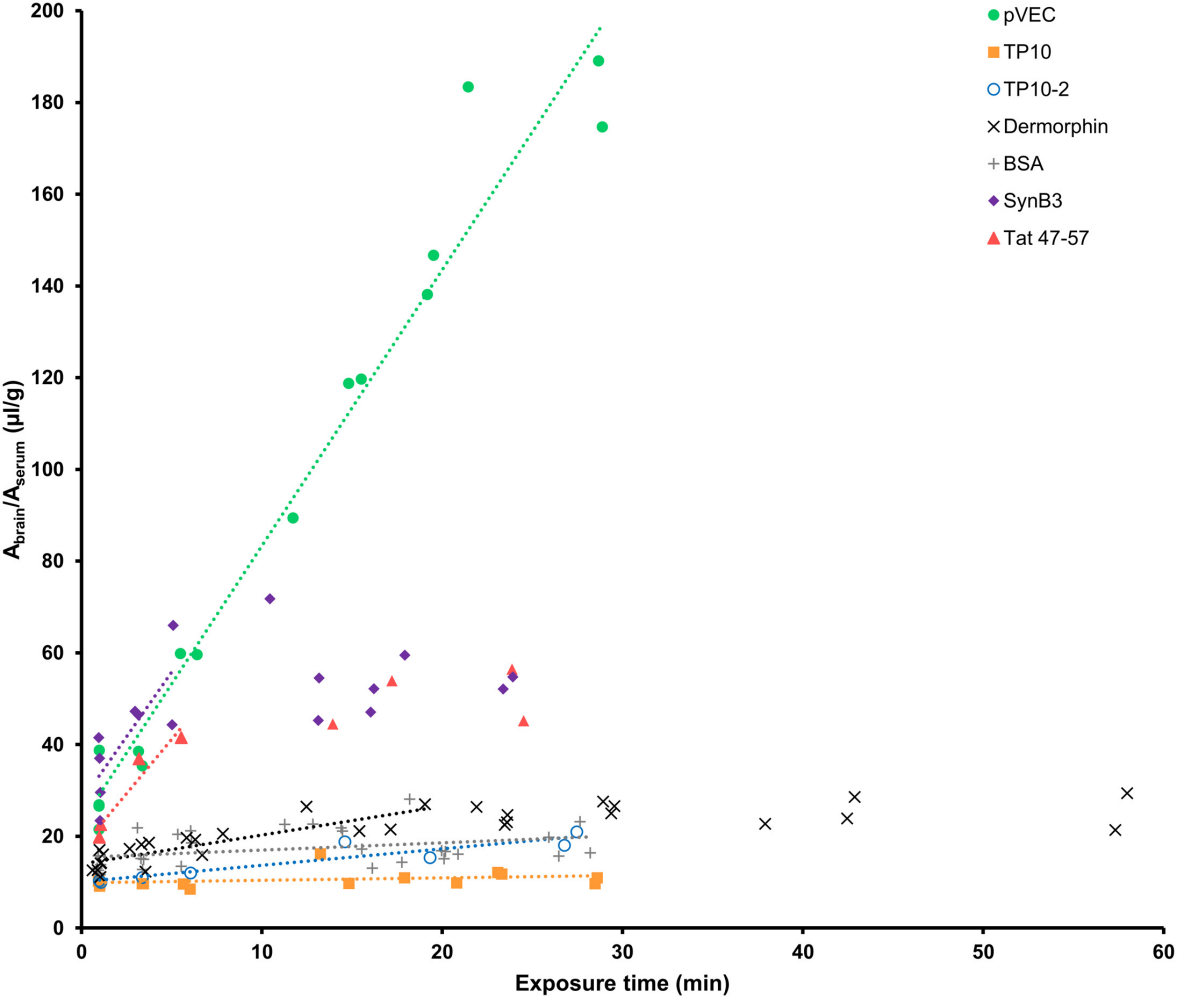
Results of the multiple time regression analysis experiment of the five CPP using the linear model.

The results of the multiple time regression (MTR) analysis indicated that the five investigated CPPs crossed the BBB to a different extent. In Fig 1, the ratio of the brain and serum radioactivity is plotted versus the exposure time and the linear part of the curve was fitted using the Gjedde-Patlak model [37–40]. The unidirectional influx rates (K_in_) and initial brain distribution volumes (V_i_) are summarized in Table 2. For pVEC, SynB3 and TP10, the MTR experiments were performed twice and their K_in_ and V_i_ were calculated by fitting all obtained data points. pVEC showed the highest brain influx rate of 6.02 μl/(g × min), followed by SynB3 and Tat 47–57 having a K_in_ of 5.63 μl/(g × min) and 4.73 μl/(g × min), respectively. In contrast, the transportan analogs showed a limited brain influx, especially TP10 that had a K_in_ close to zero (0.05 μl/(g × min)). The brain influx of TP10-2 (0.36 μl/(g × min)) was of the same magnitude as the positive control dermorphin, which is known to have a low to medium brain influx rate. The radioiodinated vascular marker BSA showed a significant influx into the brain (K_in_ = 0.16 μl/(g × min)). Bovine serum albumin has been demonstrated to show non-specific binding to cerebral capillaries of which the extent was much greater for radioiodinated than tritiated BSA, explaining its significant K_in_-value [41].

**Table 2:** Overview of the quantitative influx characteristics (± 65% confidence limits) of the five investigated CPPs based on linear and biphasic modeling of the multiple time regression analysis data.

Influx parameter	pVEC	TP10	TP10-2	SynB3	Tat 47–57	Dermorphin	BSA
**Linear Gjedde-Patlak model**							
K_in_ (μl/(g× min))	6.02 ± 0.29	0.05 ± 0.04	0.36 ± 0.06	5.63 ± 1.83	4.73 ± 1.23	0.63 ± 0.10	0.16 ± 0.07
V_i_ (μl/g)	23.13 ± 4.33	9.88 ± 0.66	10.07 ± 1.00	27.62 ± 5.58	17.43 ± 4.04	13.98 ± 0.79	15.42 ± 1.11
**Biphasic model**							
V_0_ (vascular) (μl/g)	-	-	-	10.27	10.27	10.27	-
V_g_ (tissue) (μl/g)	-	-	-	44.47 ± 7.75	34.38 ± 9.32	13.72 ± 2.17	-
K_1_ (μl/(g × min))	-	-	-	29.84 ± 8.42	13.50 ± 3.88	2.54 ± 0.50	-
K (μl/(g × min))	-	-	-	≈ 1.92e^-16^	0.27 ± 0.46	0.03 ± 0.06	-

K_in_ = Unidirectional influx rate.

V_i_ = Initial brain distribution volume.

V_0_ = Vascular brain distribution volume, experimentally determined as the brain distribution volume of radioiodinated BSA.

V_g_ = Tissue brain distribution volume.

K_1_ = Unidirectional clearance or slope of the initial phase of the brain influx curve.

K = Net clearance or slope of the plateau phase of the brain influx curve.

The ratio of the brain-to-serum activity is plotted versus the exposure time and fitted using the linear Gjedde-Patlak model. For SynB3, Tat 47–57 and dermorphin, only the linear part of the curve is fitted using the linear model.

**Fig 2:**
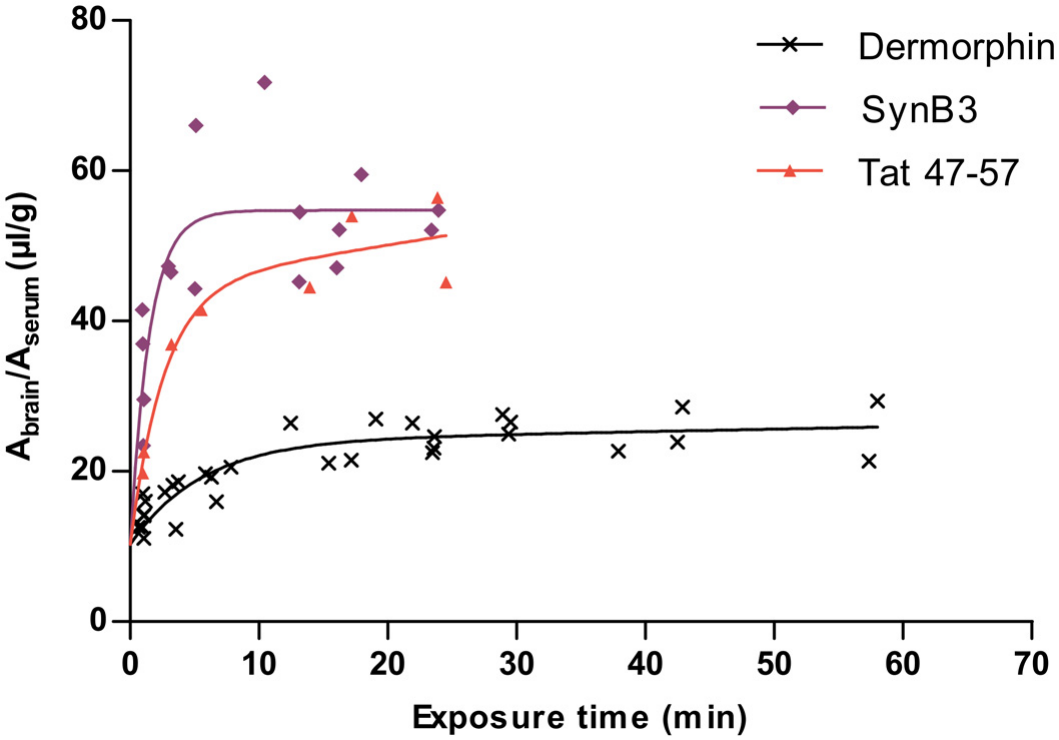
Result of the multiple time regression analysis experiment of SynB3, Tat 47–57 and dermorphin using the biphasic model. The ratio of the brain-to-serum activity is plotted versus the exposure time and fitted using the biphasic model.

The curves of the ratio of the brain and serum activity versus the exposure time of SynB3 and Tat 47–57 reached a plateau-phase after about 5 min. In Fig 2, the MTR data of these peptides were fitted using a biphasic model and is summarized in Table 2 [42]. K_1_ represents the unidirectional influx of the initial phase of curve and for SynB3 the K_1_ (29.84 μl/(g × min)) is more than twice as high as that of Tat 47–57 (13.50 μl/(g × min)). After reaching the plateau-phase, which can be explained by efflux of the peptide out of the brain and/or distribution and elimination of the peptide, the ratio of the brain and serum activity did no longer increase for these peptides: K is about 0 μl/(g × min) for SynB3 and is 0.27 ± 0.46 μl/(g × min) for Tat 47–57, which is not statistically significantly different from zero. Thus, after the initial phase characterized by high brain influx rates, the BBB transport of SynB3 and Tat 47–57 reached a plateau of no net brain clearance.

The MTR curve of pVEC did not show a plateau-phase in the 15 min time range of the performed MTR experiments. Moreover, the amount of pVEC reaching the brain, represented by the ratio of the brain-to-serum activity, greatly transcends that of the other investigated CPPs. For this CPP, the initial distribution volume, representing the effective vascular space of the peptide, was higher than that of the vascular marker radioiodinated bovine serum albumin (BSA) which was about 15 μl/g. This was also observed for SynB3 for which the V_i_ was 27.62 μl/g. For the other CPPs, the V_i_ was of the same magnitude as of radioiodinated BSA (Tat 47–57) or was significantly smaller (transportan analogs).

The capillary depletion method was used to evaluate the brain parenchymal and capillary distribution of the radiolabeled peptides after perfusion of the brain in order to remove capillary bound peptides. The parenchymal fraction was 80% for pVEC, 77% for SynB3, 79% for Tat 47–57, 85% for TP10 and 84% for TP10-2. Thus, the measured activity of the brain during the MTR experiment mainly originated from peptides present in the brain parenchyma. Only about 15 to 25% of the CPPs remained trapped by the endothelial cells.

### Brain-to-blood transport kinetics

The efflux properties of the CPPs out of the brain were investigated by measuring the brain activity after intracerebroventricular injection of the radiolabeled peptides. The efflux transfer constant k_out_ was derived from the absolute value of the slope of the natural logarithm of the brain activity versus the experimental time curve. The k_out_ of pVEC (0.10 ± 0.11 min^-1^) was not statistically significantly different from zero. All other investigated CPPs showed a statistically significant efflux out of the brain. The k_out_ calculated for SynB3 was 0.05 ± 0.01 min^-1^, for TP10 k_out_ was 0.09 ± 0.02 min^-1^, and for TP10-2 k_out_ was 0.06 ± 0.01 min^-1^. These efflux rate constants equal a half-time disappearance (t_1/2,brain_) of 15 min, 8 min and 11 min, respectively. The highest efflux rate was observed for Tat 47–57, having a k_out_ of 0.21 ± 0.08 min^-1^ or t_1/2,brain_ of 3 min. These brain half-time disappearances of less than 15 min suggest the existence of an active efflux transport system for the investigated peptides [43]. The observed efflux of SynB3 and Tat 47–57 is also consistent with the observed plateau-phase during the MTR experiment.

### Evaluation of the influx mechanism

**Fig 3:**
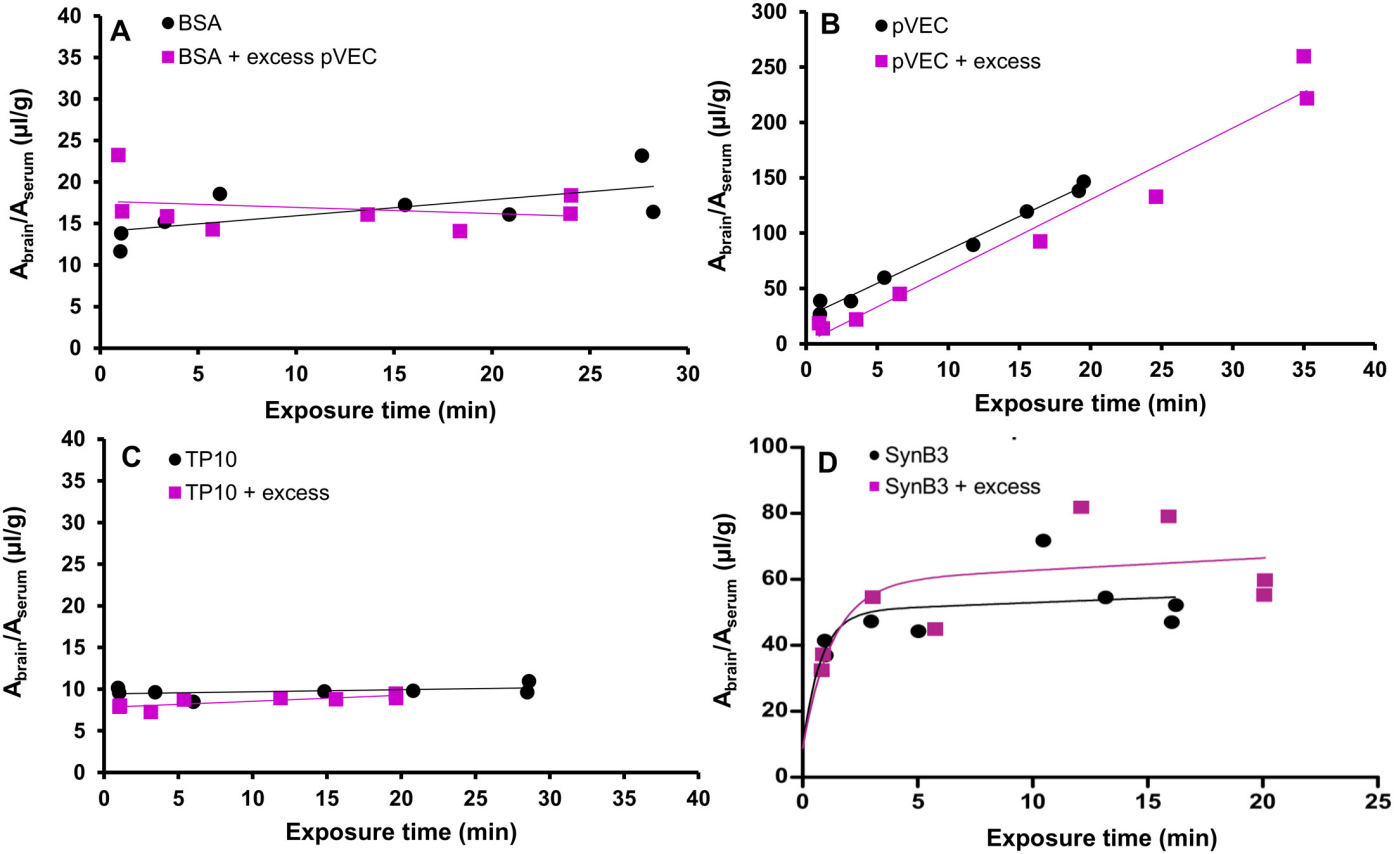
Evaluation of the BBB permeability after injection of pVEC and used brain influx mechanism of pVEC, TP10 and SynB3. (A) Evaluation of the BBB permeability after IV injection of pVEC: ratio of brain-to-serum radioactivity versus exposure time of radioiodinated BSA with (purple squares) and without (black dots) an excess dose of pVEC (20 μg). (B-D) Evaluation of the saturability of the BBB influx mechanism of pVEC, TP10 and SynB3, respectively: ratio of brain-to-serum radioactivity versus exposure time with (purple squares) and without (black dots) an excess dose of the CPP (10 μg). Data are fitted using the linear Gjedde-Patlak model, except for the data of SynB3, which are fitted using the biphasic model.

As pVEC showed an extraordinary high brain influx, the BBB transport of this peptide was further explored. We hypothesized that pVEC itself (transiently) increased the BBB permeability. To evaluate this hypothesis, a MTR experiment was performed with the radiolabeled vascular marker BSA, with and without co-injection of 20 μg of pVEC. The slopes of the curves of radioiodinated BSA and radioiodinated BSA with an excess dose of pVEC were not statistically significantly different (P > 0.05, Fig 3A). Thus, under the experimental conditions of this study, the BBB did not show an increased permeability for radioiodinated BSA after IV injection of pVEC, indicating the BBB integrity was not significantly influenced by pVEC.

Next, the saturability of the brain influx mechanism of pVEC, SynB3 and TP10, being representatives of the three different groups of CPPs observed during the BBB transport studies, was evaluated. Therefore, a MTR experiment was performed with these peptides, radiolabeled using a no-carrier added (NCA) method, with and without an excess dose of 10 μg of the unlabeled peptide. The results are shown in Fig 3B–3D: there was no statistically significant difference in unidirectional brain influx rate between the experiments of the peptide alone or when co-injected with an excess dose (P > 0.05). For SynB3, the slopes of the initial, linear part of the curve, fitted using the linear Gjedde-Patlak model, were not statistically significantly different (P > 0.05). Thus, during the performed experiments, the brain influx mechanism of pVEC, SynB3 and TP10 was not saturable. Although the K_in_ was unaffected, the excess dose of unlabeled pVEC caused a significant decrease of V_i_ from 24 μl/g to 1 μl/g, suggesting saturable binding sites on the brain endothelium, a phenomenon which was similarly observed for glucagon [44].

### Regional intra-brain distribution and tissue distribution of cell-penetrating peptides

**Fig 4:**
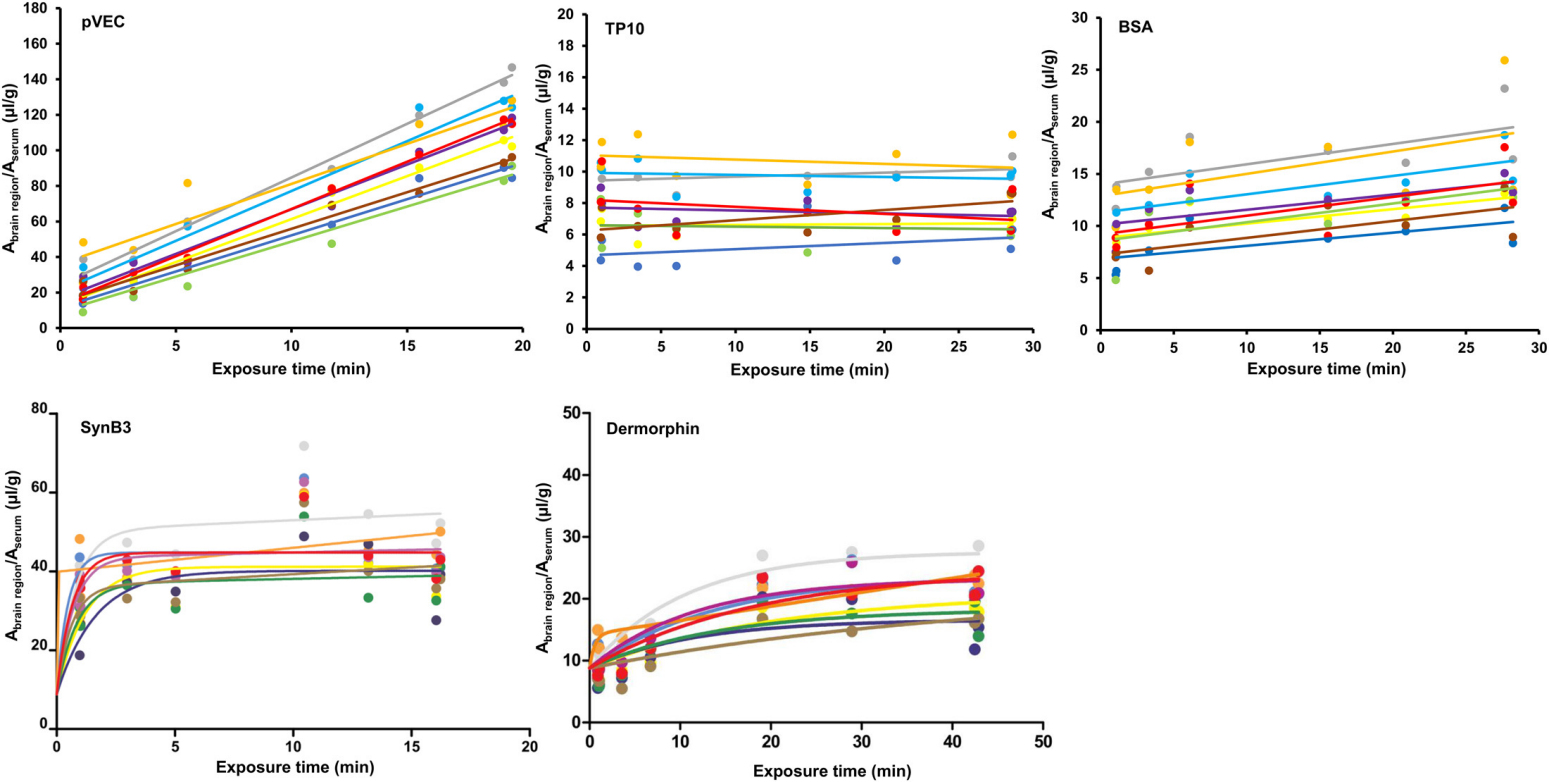
Regional variations in brain influx of pVEC, TP10, SynB3, dermorphin and radioiodinated BSA. The data of pVEC, TP10 and radioiodinated BSA are fitted using the linear Gjedde-Patlak model; the data of SynB3 and dermorphin are fitted using the biphasic model. Grey = whole brain, yellow = frontal cortex, purple = occipital + parietal cortex, light blue = cerebellum, dark blue = striatum, brown = thalamus + hypothalamus, orange = pons medulla, green = hippocampus and red = midbrain.

For pVEC, SynB3 and TP10, the regional differences in brain influx were investigated. Therefore, during the MTR experiments of these peptides, brains were dissected into eight different brain regions and their regional unidirectional influx rate K_in_ was determined using the Gjedde-Patlak model. For SynB3, TP10, as well as the controls radioiodinated BSA and dermorphin, no statistically significant difference in brain influx rates were observed between the different dissected brain regions (P > 0.05, Fig 4). The slopes, *i*.*e*. K_in_, of the initial, linear part of the curve, fitted using the linear Gjedde-Patlak model, were compared for SynB3 and dermorphin. For pVEC, the slopes of curve of the ratio of the brain-to-serum radioactivity versus the exposure time significantly differed (P < 0.05). In Table 3, the individual K_in_ and V_i_ values of the whole brain and brain regions after IV injection of pVEC are summarized. The difference in initial brain influx rates was not pronounced: the influx of pVEC was only slightly higher in the cerebellum, cortex and midbrain compared to the pons medulla, hippocampus, thalamus and hypothalamus and striatum.

**Table 3:** K_in_ and V_i_ values of the whole brain and eight brain regions of pVEC (between brackets the 65% confidence interval is indicated).

Brain region	K_in_ (μl/(g × min))	V_i_ (μl/g)
Whole brain	6.04 [5.78, 6.30]	24.37 [21.23, 27.51]
Cerebellum	5.61 [5.12, 6.10]	21.02 [15.09, 26.95]
Pons medulla	4.51 [3.87, 5.15]	36.06 [28.34, 43.78]
Frontal cortex	4.84 [4.66, 5.02]	12.93 [10.77, 15.09]
Striatum	4.07 [3.77, 4.37]	11.47 [7.81, 15.13]
Hippocampus	3.94 [3.51, 4.37]	9.33 [4.13, 14.54]
Occipital and parietal cortex	5.03 [4.73, 5.32]	16.61 [13.02, 20.19]
Thalamus and hypothalamus	4.12 [3.86, 4.38]	17.72 [11.58, 17.86]
Midbrain	5.32 [5.17, 5.46]	13.88 [12.15, 15.62]

**Fig 5:**
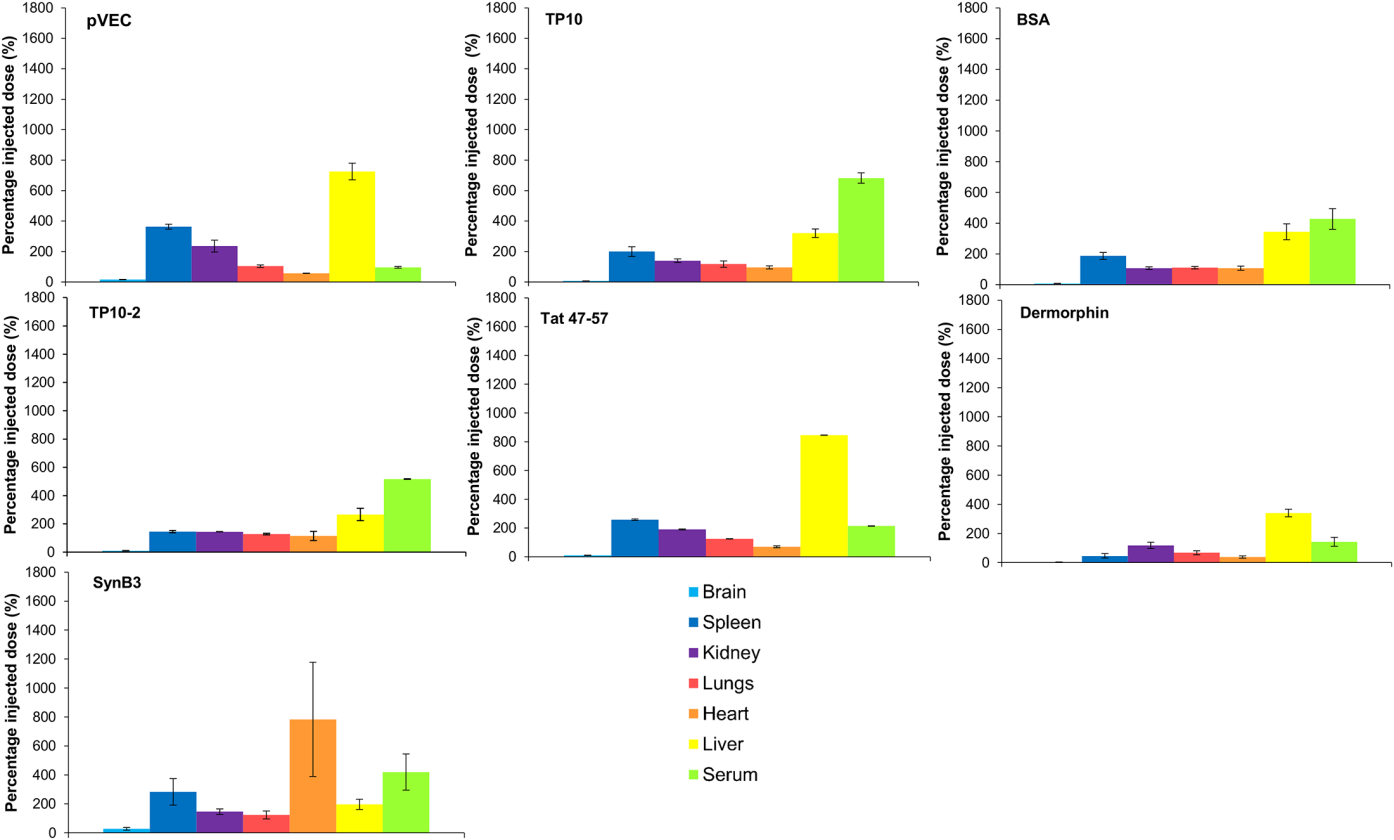
Relative tissue distribution of the radiolabeled CPPs and the controls dermorphin and radioiodinated BSA 15 min post IV injection expressed as the percentage of the injected dose (± SEM, n = 2). From the left to the right: brain (light blue), spleen (dark blue), kidneys (purple), lungs (red), heart (orange), liver (yellow) and serum (light green).

The tissue distribution of the investigated CPPs was also evaluated for mice of the 15 min time point of the MTR experiments (Fig 5). pVEC and Tat 47–57 showed a high liver distribution compared to the other tissues. The transportan analogs did also show this high liver concentration, but an even higher serum distribution was observed. SynB3 was mainly distributed to the spleen and serum and to a lesser extent to the liver. This CPP also showed a significant heart distribution. The results of the controls dermorphin and radioiodinated BSA were similar as reported in previous studies, with high liver distributions for both controls and also high serum concentrations for radioiodinated BSA [45–48]. Thus, the distribution to the different tissues varied among the different CPPs, which is also observed for other already investigated peptides [45–48].

### *In vitro* peptide stability in mouse serum and brain, kidney and liver homogenates

As data on the metabolic stability of CPPs are scarce [36,49–51], the stability of the investigated peptides was determined in mouse serum, as well as in mouse brain, liver and kidney homogenates (Table 4). In the brain and liver homogenate, the stability varied among the CPPs: in mouse brain, the half-lives ranged between 21 min (SynB3) and 176 min (TP10) and in the liver homogenate between 37 min (SynB3) and 139 min (TP10). The transportan analogs showed high serum stability, with a half-life of 22 h for TP10 and 4 h for TP10-2. In contrast, Tat 47–57, SynB3 and pVEC were not so stable in serum, having a half-life of less than 6 min. Kidney enzymes also extensively metabolized the investigated CPPs: the half-lives ranged between 5.5 min (SynB3) and 34 min (TP10). For pVEC, showing an extraordinary brain influx, the formed metabolites during serum incubation were further investigated. One metabolite was determined and identified as the pVEC peptide with the first six hydrophobic N-terminal amino acids deleted, formed by cleavage of the first N-terminal arginine-arginine bond (pVEC_7-18_), which is known to be unstable in serum (see S2 Fig) [49].

**Table 4:** Overview of the *in vitro* metabolic stability results of the five investigated CPPs.

	pVEC	TP10	TP10-2	SynB3	Tat 47–57
Tissue	Half-life (min)				
Serum	< 3	1315.8	229.4	5.5	2.7
Brain	68.4	175.9	102.3	21.4	54.3
Kidney	7.2	34.3	11.3	5.1	18.3
Liver	42.9	139.4	118.2	36.8	59.8

## Discussion

Despite the ability of CPPs to enter mammalian cells, only a few studies have fragmentarily investigated their transcellular transport characteristics. Lindgren *et al*. demonstrated for TP10 its ability to cross a Caco-2 cell layer, while penetratin passed the cell layer to a lower extent due to rapid degradation [52]. For the Tat peptide, *in vitro* transcellular delivery studies could not demonstrate the capacity of the peptide to cross the cell layers [53–55]. *In vivo* evaluation of CPP-mediated delivery of cargoes resulted in divergent outcomes. Tat-mediated delivery of neuroprotective therapeutics across the BBB in ischemia and seizure models gave promising results, but in these models the BBB is compromised [55]. For a limited set of CPPs, studies are available demonstrating their ability to mediate CNS delivery of different cargoes *in vivo* [34] (S1 Table). Currently, CPPs are investigated as possible carriers for BBB-impermeable cargoes. However, quantitative knowledge on the BBB transport characteristics of CPPs without a cargo is also needed as these peptides can be produced endogenously through metabolization of proteins and might exert biological activity. As this information is currently lacking, the *in vivo* BBB transport of five model CPPs was investigated to evaluate whether cell-penetrating properties of peptides inherently imply the ability to cross the BBB. The selected peptides constitute different clusters in the exploration of the chemical space of the CPPs and thus structurally disseminate [1] (see S1 Fig). TP10-2 only differs one amino acid from TP10, but differentiates in α-helicity and extent of cellular uptake [56]. Beside these structural variability, the model peptides also differ in cell-penetrating ability, expressed as the cell-penetrating (CP-) response: TP10 and pVEC have the highest cellular influx with a CP-response of 1.641 and 1.318, respectively, while SynB3 and Tat 47–57 show the lowest cellular penetration, having a CP-response of 0.126 and 0.309, respectively (Table 1) [1].

In this study, the selected CPPs showed quite different BBB transport properties. Tat 47–57, pVEC and SynB3 showed relatively high unidirectional influx rates (K_in_), of 4.73 μl/(g × min), 6.02 μl/(g × min) and 5.63 μl/(g × min), respectively, as obtained by fitting the MTR data using the linear Gjedde-Patlak model. On the other hand, the transportan analogs showed a very low to negligible brain influx. The blood-to-brain transport of pVEC was extraordinary high: the amount of peptide reaching the brain was much higher compared to SynB3 and Tat 47–57, with a maximal ratio of brain-to-serum radioactivity of about 180 μl/g for pVEC versus about 70 μl/g for SynB3. This high brain influx was not caused by an increase in BBB permeability, as co-injection of radioiodinated BSA and pVEC did not result in an increased brain entry of the vascular marker. Using the capillary depletion method, it was demonstrated that the peptides effectively crossed the BBB with a parenchymal fraction of about 80% for all peptides. For SynB3, TP10 and the controls dermorphin and radioiodinated BSA, no differences in influx between the eight dissected brain regions were observed. For pVEC, the K_in_ values of the dissected brain regions statistically differed, but the observed regional difference was not pronounced compared to other peptides such as amylin and insulin showing clear regional variations in brain influx [57]. It is unlikely that alterations in cerebral blood flow due to the changing local metabolic demand explain the observed differences in regional brain uptake as this only affects the uptake of compounds showing a very rapid brain influx [57–59]. The BBB influx (K_in_) of pVEC, TP10 and SynB3 was not saturated when peptides were co-injected with an excess dose of 10 μg of unlabeled peptide. For pVEC, the V_i_ significantly decreased suggesting the presence of saturable binding sites, similarly as observed for glucagon. These saturable binding sites may include receptors or enzymes located at the brain capillary endothelium [44].

In contrast to pVEC, both SynB3 and Tat 47–57 showed a biphasic BBB influx behavior: after an initial sharp increase, the MTR-curve reached a plateau-phase, resulting in a non-significant net brain clearance of these peptides. This plateau-phase can at least partly be explained by the significant efflux of SynB3 (k_out_ = 0.05 min^-1^) and Tat 47–57 (k_out_ = 0.21 min^-1^) out of the brain. Except for pVEC, the other CPPs showed a significant efflux as well with a k_out_ of 0.09 min^-1^ and 0.06 min^-1^ for TP10 and TP10-2, respectively. The brain half-time disappearances of the CPPs suggest the existence of an active efflux transport system [43].

All investigated CPPs were mainly distributed to the liver and serum, which was also previously demonstrated for pVEC and TP10 [49]. For pVEC and Tat 47–57, a very high liver concentration was observed compared to the other tissues, which can indicate metabolization, but also uptake of the peptides and their metabolites in hepatocytes. The transportan analogs and SynB3 showed high serum distribution that can be explained either by protein binding or high serum stability and the latter was confirmed during the *in vitro* metabolic stability study of the transportan analogs where a serum half-life of 22 h was calculated for TP10 and of 4 h for TP10-2. The other CPPs were not stable in serum, having half-lives of less than 6 min. Thereby, it cannot be excluded that radiolabeled metabolites of these CPPs contribute to the observed brain radioactivity during the evaluation of the blood-to-brain transport. During *in vivo* metabolic stability studies, metabolites present in serum as well as in brain tissue can be identified, providing the full picture of which peptides do actually cross the BBB. This information is valuable during structure-property relationship studies for BBB transport of CPPs, or peptides in general. The low serum stability originates from the presence of arginine-arginine bonds, which are absent in the sequence of the transportan analogues [49]. The low stability was already reported for Tat 47–57 [50], as well as for pVEC, for which a rapid C-terminal lysine (Lys^18^) cleavage was demonstrated when incubated in human serum [51]. In contrast, another study demonstrated a (human) serum half-life of a few hours by evaluating the activity of the ^68^Ga-DOTA labeled pVEC [49]. In this *in vitro* study, we could not demonstrate the formation of this Lys^18^-cleaved pVEC metabolite when incubated in mouse serum. Instead, we identified a metabolite being pVEC from which the first six hydrophobic N-terminal amino acid residues were cleaved off (pVEC_7-18_). A structure-activity relationship study revealed that these, cleaved-off N-terminal hydrophobic residues are crucial for the cellular uptake properties, suggesting that the found metabolite pVEC_7-18_ is not cell-penetrating [60]. Thus, both pVEC and pVEC_7-18_, having similar cationic nature, could contribute to the measured brain activity during the MTR experiment of pVEC, as both contain a radiolabeled tyrosine residue. Once the brain tissue was reached, the mouse brain half-life indicates the peptides remain sufficiently stable to allow further distribution in the brain parenchyma.

**Fig 6:**
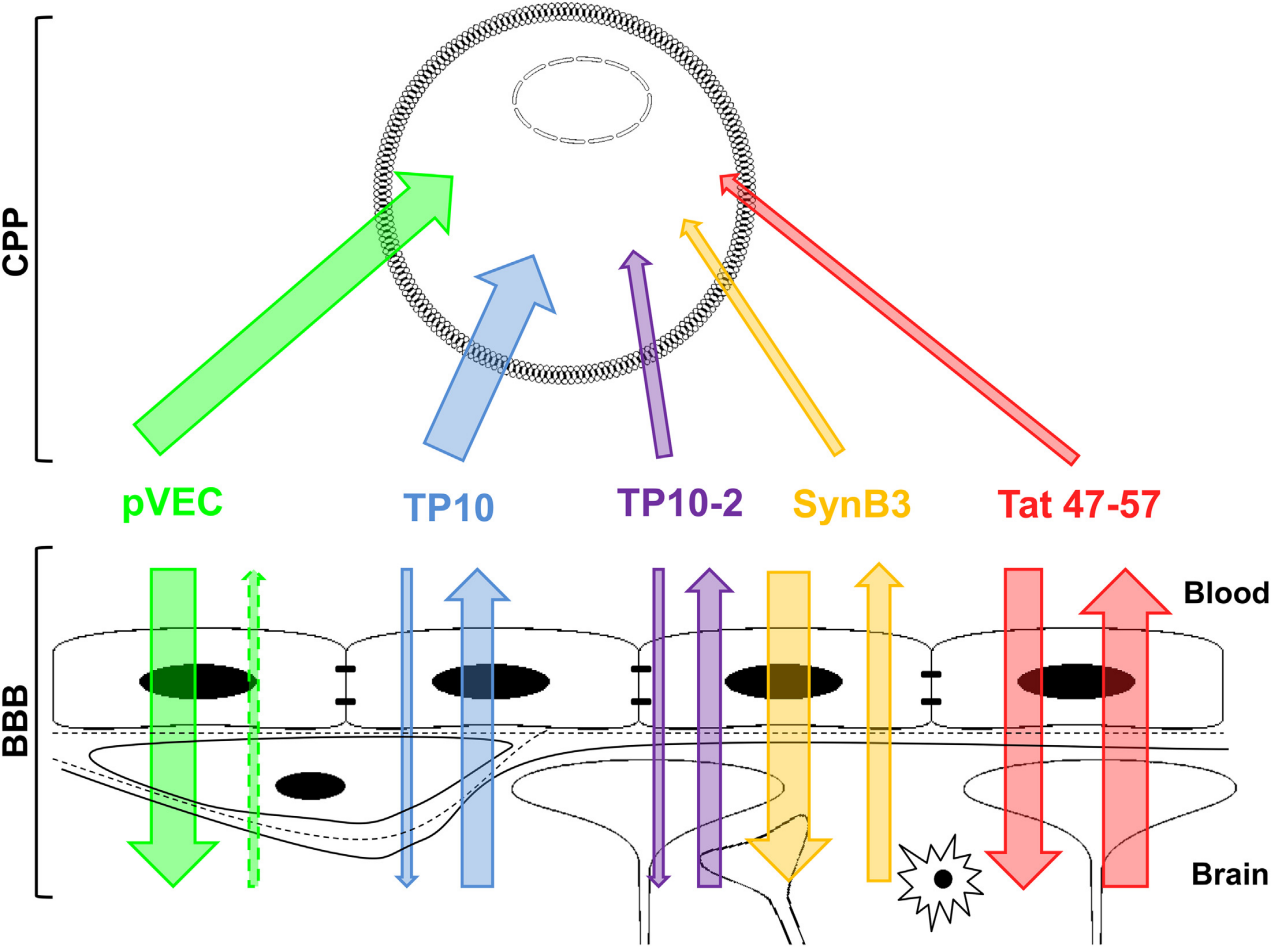
Schematic overview of the relationship between cell-penetrating and BBB-penetrating properties of the five investigated CPPs. The thickness of the arrows indicates the extent of influx and/or efflux.

Based on the BBB transport results, three groups of CPPs could be distinguished. The cationic-amphipathic peptide pVEC constitutes the first group, which showed a rapid brain influx, resulting finally in relatively high ratio of brain-to-serum radioactivity. SynB3 and Tat 47–57, both short cationic CPPs, form the second group and had relatively high initial brain influx rates, but their brain influx shows a biphasic behavior. The third group consists of the transportan analogs, TP10 and TP10-2, which showed no to slow brain influx, respectively. The first two groups are composed of arginine-rich peptides, while the transportans only contain lysine-residues in their sequences and have a much lower charge density of 19% versus 44%, 50% and 73% of pVEC, SynB3 and Tat 47–57, respectively (Table 1). Thus, arginine-rich CPPs seem to more effectively and rapidly influx the BBB in the investigated experimental time period. The BBB influx properties of pVEC, SynB3 and Tat 47–57 are also superior to other peptides already investigated for their BBB transport characteristics: using our recently proposed classification method [61], these peptides show a very high brain influx (class 5), while TP10 and TP10-2 have a very low (class 1) and low (class 2) brain influx, respectively. These findings were not expected based on the cell-penetrating properties of these CPPs, quantitatively expressed as the CP-response [1]. TP10 had the highest CP-response (1.641), but the poorest BBB influx characteristics. In contrast, medium cell-penetrating properties were attributed to SynB3 and Tat 47–57, but high BBB influx was concluded [1]. Our data indicate that CPPs selectively cross the BBB and that their brain influx behavior cannot be directly positively correlated with their cell-penetrating properties (Fig 6). A possible explanation can be found in their differences in cellular influx mechanism and secondary structure at the membrane interface. For SynB3, an endocytosis-dependent mechanism is described, which is initiated after an electrostatic interaction [62]. The uptake of Tat 47–57 is endocytosis-driven as well, but starting from a certain threshold concentration, the peptide directly penetrates into the cell [3,62–64]. The use of an endocytosis-dependent and -independent mechanism has been demonstrated for pVEC [3,60,65,66]. This peptide is derived from the murine vascular endothelial (VE)-cadherin, located in the adherens junctions between the vascular endothelial cells, and constitutes the 13 cytosolic amino acids closest to the membrane and five amino acids from the C-terminus of the transmembrane region (615–632) [19]. As already mentioned, these five hydrophobic residues, located at the N-terminus of the pVEC sequence, appear to be crucial for cellular uptake and directly interact with the plasma membrane [60]. The cellular interaction of pVEC, SynB3 or Tat 47–57 does not cause any membrane disturbances [60,62]. This corroborates with our results where we could not demonstrate that pVEC increased the BBB permeability, as the influx of radioiodinated BSA did not augment after co-injection with pVEC. For TP10, the used cell-penetrating mechanism remains controversial: Padari *et al*. described an endocytosis-driven mechanism, while more recent studies ascribe a pore-forming mechanism [67–69].

The investigated peptides also have a different secondary structure at the membrane interface: when in solution, all peptides have a random coil structure, but at the membrane interface, pVEC adopts a β-sheet structure and TP10 becomes α-helical, while SynB3 and Tat 47–57 remain random coiled [56,70]. Overall, the investigated CPPs differ essentially in the presence or absence of arginine residues, thus their cationic nature (chemical properties), in their secondary structure at the membrane interface (physicochemical properties) and inherently, in the cellular uptake mechanism (biological properties).

Currently, four different BBB transport mechanisms are described for peptides. The non-saturable mechanism involves passive diffusion across the BBB, which is mainly used by lipophilic peptides. The saturable mechanisms include receptor-mediated transcytosis, involving a specific receptor, carrier-mediated transcytosis, following an interaction with a transporter located at the endothelial cell surface, and adsorptive-mediated transcytosis, which is a non-specific transport mechanism triggered by an electrostatic interaction between positively charged peptides and the negatively charged cellular membrane. For SynB3, it was already proposed that the adsorptive-mediated transcytosis mechanism was used to cross the BBB [36]. As the BBB transport data indicate that a high charge density derived from arginine residues are favorable for BBB influx, it is assumed that an initial electrostatic interaction with the negatively charged glycocalyx and phospholipid head groups triggers the transport across the BBB, followed by penetration or endocytosis. Under our experimental conditions, we could not demonstrate that pVEC, SynB3 nor TP10 used a saturable transport mechanism to cross the BBB, pointing to the passive diffusion mechanism.

## Conclusion

The BBB transport properties of five structurally different model CPPs, showing a variable extent of cellular penetration, were investigated. SynB3, Tat 47–57 and pVEC showed relatively high (initial) brain influx rates, while the influx of TP10 and TP10-2 was low. The CPPs use a non-saturable influx mechanism and do not cause a (transient) increase in BBB permeability, as demonstrated for pVEC. Except for pVEC, all peptides showed a significant efflux out of the brain, which partly explains the biphasic behavior of SynB3 and Tat 47–57. Our BBB transport results indicate that CPPs selectively cross the BBB and thus cell-penetrating properties of peptides do not imply BBB-penetrating ability.

## Materials and Methods

### Peptide quality

TP10 and TP10-2 were purchased at Caslo ApS (Lyngby, Danmark); pVEC, SynB3 and Tat 47–57 at LifeTein LLC (Somerset, USA) and the positive control dermorphin at Bachem (Bubendorf, Switzerland) and Hanhong group (Shanghai, China). The peptide purity was verified by (U)HPLC-analysis and estimated ≥ 95% [71].

### Radioiodination and purification of peptides and BSA

SynB3, Tat 47–57, dermorphin and albumin (BSA) (Merck KGaA, Darmstadt, Germany) were radiolabeled using the Iodo-Gen method. A previously established procedure was used [45], but in case of Tat 47–57 and dermorphin, a 1 μmol/ml sodium iodide carrier solution was used. The peptides were iodinated by transfer of the iodonium solution to 50 μl of a 1 μmol/ml peptide solution. TP10, TP10-2 and pVEC were radiolabeled using the chloramine-T method [72]. Briefly, to 50 μl of a 1 μmol/ml peptide solution (TP10 and TP10-2) or 110 μl of a 1 mg/ml pVEC solution, dissolved in 0.1% formic acid in 95:5 water:acetonitrile, 20 μl of a 3.75 μmol/ml sodium iodide in aqueous 0.1% (m/V) formic acid (TP10 and TP10-2) solution or 20 μl of a 2.5 μmol/ml sodium iodide in 25 mM phosphate buffer pH 8.5 (pVEC) was added. Then, a volume containing 1 mCi of Na^125^I (Perkin Elmer, Zaventem, Belgium) was transferred to this solution, followed by 30 μl of a 0.5 mg/ml chloramine-T solution in 25 mM phosphate buffer (pH 7.4 (TP10 and TP10-2) or pH 8.5 (pVEC)). The iodination reaction proceeded during 40 s, followed by the addition of 30 μl of a 1 mg/ml sodium metabisulfite in 25 mM phosphate buffer (pH 7.4 (TP10 and TP10-2) or pH 8.5 (pVEC)) to stop the iodination reaction. For evaluation of the used influx mechanism, pVEC, SynB3 and TP10 were radiolabeled using a no-carrier added protocol, in which the sodium iodide solution was replaced by its solvent.

After radiolabeling, the iodinated peptides were fractionated using an HPLC-UV apparatus (LaChrom Elite, Hitachi, Tokyo, Japan) equipped with a radioactivity detector (Berthold Technologies GmbH & Co. KG, Bad Wildbad, Germany) and fraction collector (Gilson International BV, Den Haag, The Netherlands), in line with the HPLC waste. For separation, a Vydac Everest C_18_ column (250 × 4.6 mm, 5 μm particle size; Grace, Lokeren, Belgium) was coupled to the HPLC system. Mixtures of water and acetonitrile acidified with 0.1% (m/V) formic acid or 0.1% (m/V) trifluoroacetic acid (SynB3 and Tat 47–57) were used to create appropriate gradients for separation of peptides and their iodinated forms. After fractionation, the fractions containing the radiolabeled peptides were selected and evaporated using a N_2_ flow. For BSA, after radio-iodination, 500 μl of phosphate buffer (130 mM, pH 7.4) was added and the solution was filtered over a silver filter (Sterlitech, Kent, USA). Before each experiment, a quality control of the iodinated peptide stock was performed.

### *In vivo* experiments in mice

Female, ICR-CD-1 mice (Harlan Laboratories, Venray, The Netherlands), aged 7 to 10 weeks and weighing 28–36 g, were used during the *in vivo* BBB transport experiments. All animal experiments were performed in accordance with the Ethical Committee principles of laboratory animal welfare. The protocol was approved by the Ethical Committee of the Faculty of Veterinary Science of Ghent University (Permit Numbers: 2009–052 and 2014–128). Prior to experiments, mice were anesthetized by intraperitoneal injection of a 40% urethane solution (3 g/kg) (Sigma-Aldrich, Diegem, Belgium).

### Evaluation of blood-brain barrier influx

The protocol of the multiple time regression (MTR) analysis to evaluate whether the peptides cross the BBB is described in detail elsewhere [45]. Shortly, 200 μl of the radioiodinated CPP solution (30000 cpm/μl) was injected into the right jugular externalis vein of anesthetized ICR-CD-1 mice, which were then decapitated at 1, 3, 5, 10, 12.5 and 15 min post injection, with first and last time points performed in duplicate, after which brains were collected and measured for radioactivity using a gamma counter (Wallac Wizard 1470, Perkin Elmer). Shortly before decapitation, blood was collected from the left carotid artery, which was centrifuged (10000 *g*, 15 min at 21°C) in order to measure the radioactivity of blood and serum. Dermorphin and radioiodinated BSA were used as positive and negative control, respectively. The blood-to-brain entry during the experimental period of 15 min was evaluated by plotting the ratio of brain (A_m_(T)) and serum activity (C_p_(T)), corrected for the brain weight and serum volume, respectively, versus the exposure time (Θ), expressing the theoretical steady state serum level of the iodinated peptide at a given serum concentration [37,73]. The exposure time is calculated as the integral of the arterial serum radioactivity over time divided by the radioactivity at time t. The integral is calculated through the trapezoidal rule of the log-transformed data [73]. In the linear part of the curve, assuming a two-compartmental BBB model, data can be fitted using a linear model from which the unidirectional influx rate (K_in_), also referred to as unidirectional blood to brain clearance K_1_ [46], and the initial brain distribution volume (V_i_) can be determined using the following equation according to Gjedde and Patlak [37–40]:

$$\frac{{\text{A}}_{\text{m}}\text{(T)}}{{\text{C}}_{\text{p}}\text{(T)}}=\frac{{\text{A}}_{\text{brain}}\text{(T)}}{{\text{A}}_{\text{serum}}\text{(T)}}={\text{K}}_{\text{in}}\cdot \Theta +{\text{V}}_{\text{i }}\text{ with }\Theta \;=\;\overset{\text{T}}{\underset{\text{0}}{\int }}\frac{{\text{C}}_{\text{p}}\text{(t) }\cdot \text{ dt}}{{\text{C}}_{\text{p}}\text{(T)}}$$ (1)

If during the experimental time frame the curve deviates from linearity due to a significant efflux out of the brain resulting in a transition from unidirectional to net transfer, the following expansion of the Gjedde-Patlak plot, a model of biphasic blood-brain transfer as derived from [42], was used to fit the uptake:

$$\frac{{\text{A}}_{\text{m}}\text{(T)}}{{\text{C}}_{\text{p}}\text{(T)}}=\frac{{\text{A}}_{\text{brain}}\text{(T)}}{{\text{A}}_{\text{serum}}\text{(T)}}\text{K}\cdot \Theta +{\text{V}}_{\text{g}}\left(\text{1}-{\text{e}}^{\left(-\Theta \left(\frac{{\text{K}}_{\text{1}}-\text{K}}{{\text{V}}_{\text{g}}}\right)\right)}\right)+{\text{V}}_{0}\;\overset{\text{K}=\text{0}}{≅}{\text{V}}_{\text{g}}\left(\text{1}-{\text{e}}^{\left(-\Theta \left(\frac{{\text{K}}_{\text{1}}}{{\text{V}}_{\text{g}}}\right)\right)}\right)+{\text{V}}_{\text{0}}$$ (2)

with K_1_ is the unidirectional clearance, K is the net clearance, V_g_ the brain tissue distribution volume, and V_0_ the vascular brain distribution volume, experimentally determined as the brain distribution volume of radioiodinated BSA [46,74].

To evaluate whether the BBB remains intact after intravenous injection of the CPPs, a MTR experiment was performed for radioiodinated BSA with and without an excess dose of 20 μg of pVEC. This dose of 20 μg was determined based on the amount of peptide injected during a MTR experiment estimated based on the specific activity and taking into account the amount of non-radioactively iodinated peptide present in the injected dose. A MTR experiment with and without co-injection of an excess dose of 10 μg of unlabeled peptide was used to investigate whether the CPPs use a saturable or non-saturable transport mechanism to cross the BBB. During this experiment, peptides are radiolabeled using a no-carrier added method in order that only radioactively labeled peptide was injected.

### Tissue distribution after IV injection

At the 15 min time points of the MTR experiment, six tissues, *i*.*e*. spleen, kidneys, lungs, heart and liver, were collected immediately after decapitation, and weighed and measured in a gamma counter. The percentage of the injected dose for each isolated tissue was calculated as follows:

$$\text{\% injected dose }=\;\frac{{\text{A}}_{\text{tissue}}/{\text{w}}_{\text{tissue}}}{{\text{A}}_{\text{IV}}{\;}_{\text{injected}}/{\text{w}}_{\text{mouse}}}$$ (3)

with A_tissue_ and A_IV injected_ the measured radioactivity of the isolated tissue and the radioactivity of 200 μl of MTR stock solution, respectively, and w_tissue_ and w_mouse_ the weight of the considered tissue and injected mouse, respectively.

### Regional variation in brain influx between different brain regions

After measuring the whole brain radioactivity, the brains collected during the MTR experiment of pVEC, SynB3 and TP10, as well as of the controls dermorphin and radioiodinated BSA, were dissected into eight brain regions in order to evaluate regional variations in brain influx: (1) cerebellum, (2) medulla oblongata, (3) frontal cortex, (4) striatum, (5) hippocampus, (6) thalamus + hypothalamus, (7) midbrain and (8) occipital + parietal cortex, including “rest of brain”. Different dissected brain regions were weighed and measured in the gamma counter. The unidirectional influx and initial distribution volume of the iodinated peptides for the different brain regions are determined using Eq 1.

### Peptide distribution to brain parenchyma and capillaries

The capillary depletion method was used to evaluate the distribution of the peptide between the brain parenchyma and capillaries [75,76]. In summary (see Materials and Methods in [45]), 200 μl of a radiolabeled peptide solution (10000 cpm/μl) was injected in the jugular externalis vein of two anesthetized ICR-CD-1 mice. Ten minutes after IV injection, mice were decapitated and brains were collected and measured for radioactivity. Prior to decapitation, blood was collected from the abdominal aorta followed by intracardial perfusion of the brain using 20 ml of lactated Ringer’s buffer after clamping the aorta and severing the jugular veins. Then, brain was homogenized with 0.7 ml of ice-cold capillary buffer and 1.7 ml of 26% ice-cold dextran solution in capillary buffer. The resulting homogenate was weighed and centrifuged at 5400 *g* for 30 min at 4°C, resulting in a pellet, *i*.*e*. capillaries, and supernatant, *i*.*e*. parenchyma and fat tissue, which were weighed and measured in the gamma counter. The radioactivity of blood and serum, obtained by centrifuging blood at 10000 *g*, 15 min at 21°C, were measured as well. Compartmental distribution was calculated as follows:

$${\text{Fraction}}_{\text{capillaries or parenchyma }}\text{(\%)}=\frac{\frac{{\text{A}}_{\text{capillaries or parenchyma}}}{{\text{A}}_{\text{serum}}}}{\frac{{\text{A}}_{\text{capillaries}}}{{\text{A}}_{\text{serum}}}+\frac{{\text{A}}_{\text{parenchyma}}}{{\text{A}}_{\text{serum}}}}\times 100$$ (4)

### Brain-to-blood transport

For evaluation of the efflux of the peptides out of the brain, 1 μl of a radioiodinated peptide solution (25000 cpm/μl) was injected in the lateral ventricle of anesthetized ICR-CD-1 mice at a speed of 360 μl/h for 10 s using a syringe pump (KDS100, KR analytical, Cheshire, UK). At 1, 3, 5, 10, 12.5 and 15 min post injection, mice were decapitated and the brains were isolated and measured in the gamma counter. Prior to decapitation, blood was collected from the abdominal aorta, which was subsequently centrifuged (10 000 *g*, 15 min at 21°C) to obtain serum of which the radioactivity was measured. The brain half-time disappearance (t_1/2,brain_) was calculated from the linear regression of the natural logarithm of the residual radioactivity in brain versus time as follows:

$${\text{t}}_{\text{1/2,brain}}=\frac{\text{ln(2)}}{{\text{k}}_{\text{out}}}$$ (5)

with k_out_ defined as the efflux rate constant calculated as the absolute value of the slope of the linear regression, applying first order kinetics [45,77]. An extensive description of the protocol can be found elsewhere [45].

### *In vitro* metabolic stability

The *in vitro* metabolic stability of Tat 47–57, SynB3, pVEC, TP10 and TP10-2 was determined in mouse serum and mouse brain, liver and kidney homogenates according to pre-established protocols [45,78,79]. Prior to use, the protein content of each homogenate was determined using the Pierce Modified Lowry Protein Assay method (Thermo Scientific) to generate a stock solution with a protein concentration of 0.6 mg/ml by dilution with Krebs-Henseleit buffer (pH 7.4, Sigma-Aldrich). In brief, 100 μl of a 1 mg/ml peptide solution, dissolved in Krebs-Henseleit buffer (pH 7.4) was added to 900 μl of tissue extract or serum (500 μl of tissue homogenate or serum + 400 μl of Krebs-Henseleit buffer (pH 7.4) = 300 μg of protein in total) and incubated at 37°C while shaking at 750 rpm. Aliquots of 100 μl were sampled after 0, 15, 30, 60, 90 and 120 min of incubation into tubes containing an equal volume of aqueous TFA (1% m/V). For the serum samples of SynB3 and Tat 47–57, aliquots were taken as well at 2, 4, 6, 8, 10 and 12 min (in a separate experiment) and for TP10 and TP10-2 at 2, 5 and 10 min. After sampling, enzymatic activity was terminated by additional heat inactivation at 95°C for 5 min, followed by cooling the samples in an ice bath for 30 min. Centrifugation at 20 000 *g* for 5 min at 5°C yielded a clear supernatant ready for HPLC-UV analysis. For the analysis of the serum samples of TP10 and TP10-2, a more extensive sample preparation was required to remove interfering compounds. Therefore, a solid phase extraction (SPE) protocol using an Oasis^®^ WCX μelution plate (Waters, Zellik, Belgium) was used prior to HPLC-UV analysis. In the Supporting Information, the protocol of the SPE extraction procedure is described (S1 Text). Blank control solutions were prepared as described above, but without adding the peptide. To confirm chemical stability and mass balance, control reference solutions without tissue homogenate or with a prior heat inactivation were analyzed as well.

The HPLC-UV system consisted of a Waters Alliance 2695 separation module and a Waters 2996 photodiode array (PDA) detector (detection from 190–400 nm, quantification at 215 nm), fitted with Empower 2 software for data handling (Waters). For each sample, 20 μl was injected and separated on a Vydac Everest C_18_ column (SynB3 and Tat 47–57) or Prevail Organic Acid (pVEC, TP10 and TP10-2) column (250 × 4.6 mm, 5 μm particle size) both from Grace at 1 ml/min in an oven set at 30°C. Appropriate gradients for separation of the peptides and their metabolites were created by mixtures of water (0.1% trifluoroacetic acid m/V) and acetonitrile (0.1% trifluoroacetic acid m/V). The half-life was determined as:

$${\text{t}}_{\text{1/2}}=-\frac{\text{ln(2)}}{\text{slope}}$$ (6)

with the slope derived from the curve of the natural logarithm of the percentage of the amount at the start of the incubation, *i*.*e*. t = 0 min, versus time.

For identification of the formed metabolites during incubation of pVEC in mouse serum, serum samples obtained at 0 min and 15 min post injection were injected into an Acquity H-class UPLC^®^ apparatus consisting of a quaternary solvent manager, an automatic sample injection system, combined with a flow through needle, a column heater and an ultra-performance LC PDA detector, with Empower 3 FR 2 software for data acquisition (all from Waters). An Acquity BEH C18 300 Å column (2.1 mm × 100 mm, 1.7 μm, Waters), thermostated at 30°C, was selected for separation using the same mobile phase as above. The eluting mobile phase was split towards both the PDA and a QDa detection system (ratio 10/1), consisting of an Acquity isocratic solvent manager and an Acquity QDa detector (both from Waters), equipped with an electrospray ionization (ESI) interface. The fraction going to the QDa was diluted with 40/10/50 (V/V/V) water/propionic acid/2-propanol at a flow rate of 0.35 ml/min. The QDa detector was operated in positive ion mode with the ESI capillary voltage set at +0.8 kV and the cone voltage at 15 V. The probe temperature was 600°C. A full mass spectrum between *m/z* 100 and 1250 was acquired at a sampling rate of 2.0 spectra/s.

### Statistics

Generally, regression lines were computed using the least squares method. Regression lines of the ratio of the brain-to-serum radioactivity versus the exposure time obtained during the evaluation of the regional brain distribution and the used brain influx mechanism, were statistically compared using the Prism 6 software (GraphPad, La Jolla, USA). If the calculated P-value was greater than 0.05, the slopes were not statistically significantly different.

## Supporting Information

S1 Fig Chemical diversity of selected model CPPs [1]. (PDF)

S2 Fig Identification of metabolite of pVEC formed during incubation in mouse serum. (PDF)

S1 Table Overview literature studies describing brain delivery by cell-penetrating peptides. (PDF)

S1 Text Oasis^®^ WCX SPE sample preparation procedure of the serum samples of TP10 and TP10-2. (PDF)
