# Supplementary figures and images for: Cell-Penetrating Peptides Selectively Cross the Blood-Brain Barrier In Vivo
Source: PLoS One. 2015 Oct 14;10(10):e0139652. doi: 10.1371/journal.pone.0139652 (PMC4605843; doi:10.1371/journal.pone.0139652)

**S1 Fig. Chemical diversity of selected model CPPs [1].**

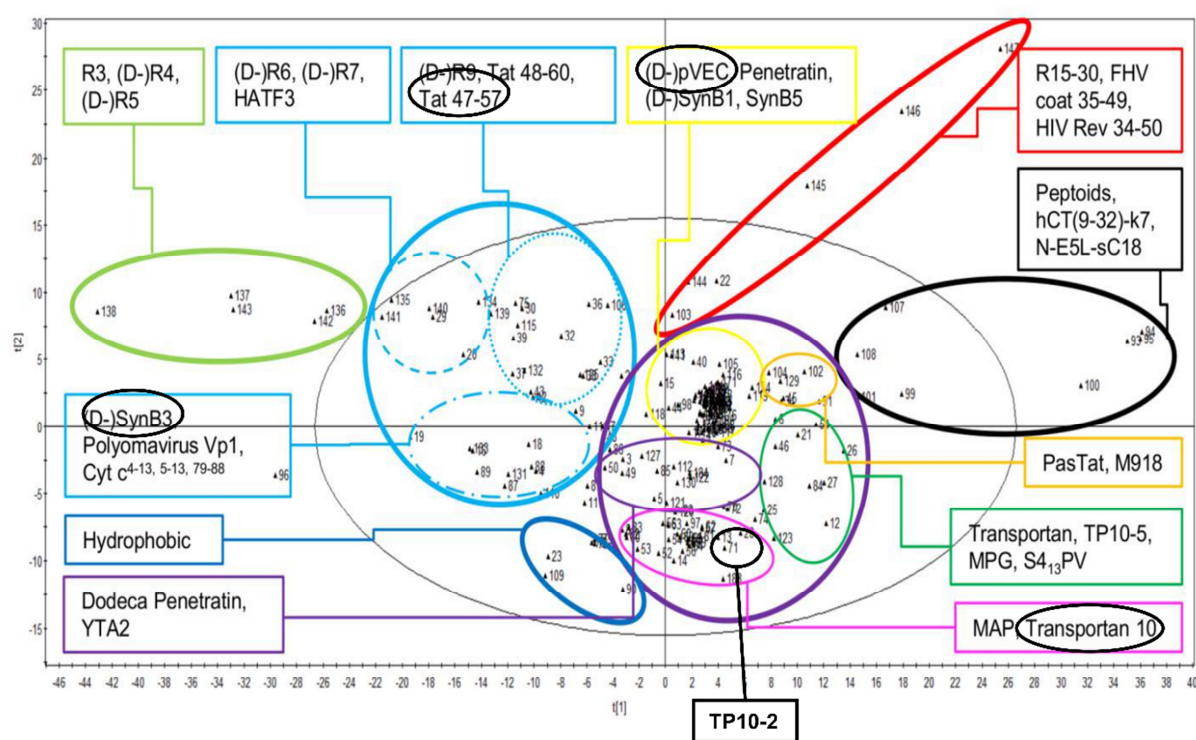

Supplement: S1 Fig — (PDF) [file pone.0139652.s001.pdf]

**S2 Fig. Identification of metabolite of pVEC formed during incubation in mouse serum.**

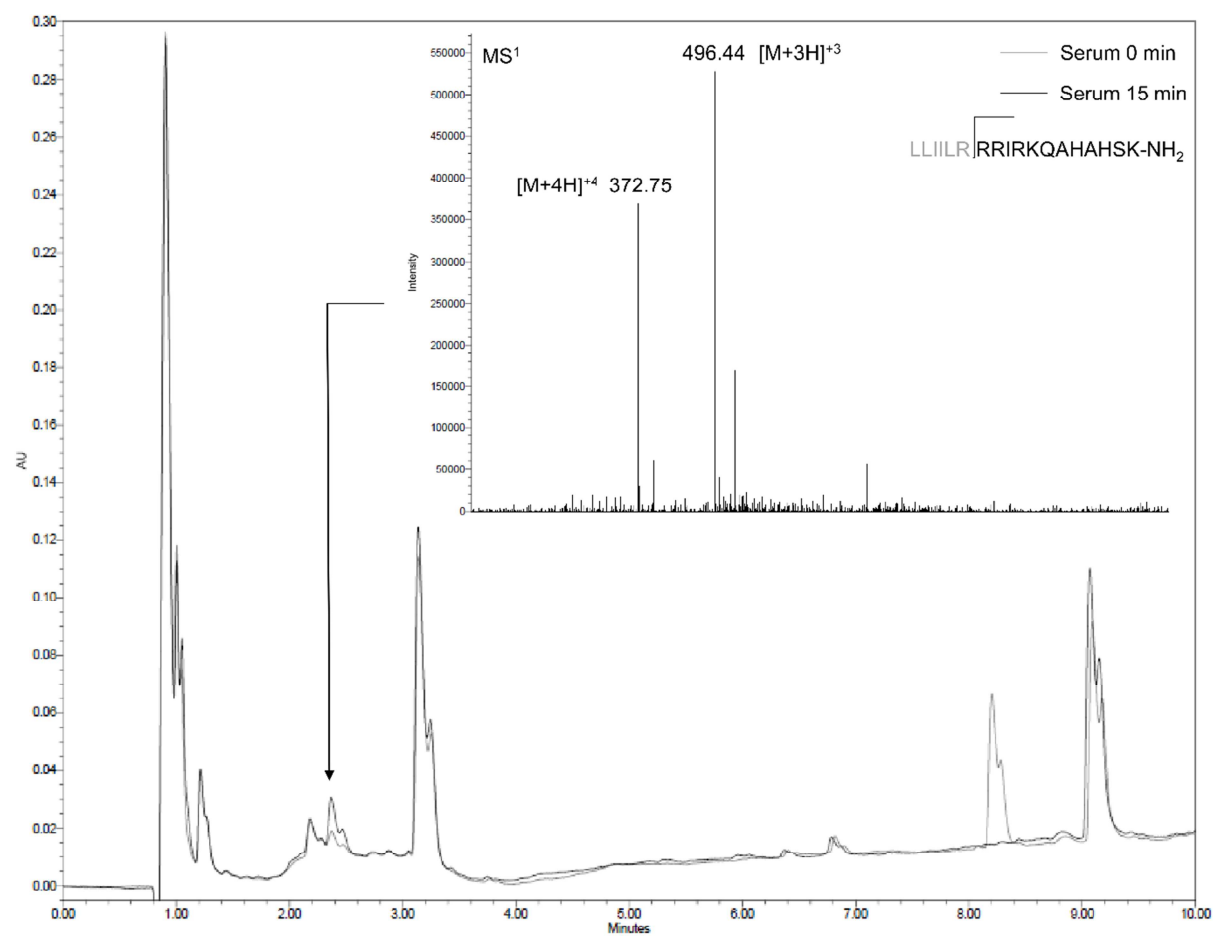

Supplement: S2 Fig — (PDF) [file pone.0139652.s002.pdf]
