# Supplementary material for: Cell-Penetrating Peptides Selectively Cross the Blood-Brain Barrier In Vivo
Source: PLoS One. 2015 Oct 14;10(10):e0139652. doi: 10.1371/journal.pone.0139652 (PMC4605843; doi:10.1371/journal.pone.0139652)
Supplement: S1 Table — (PDF) [file pone.0139652.s003.pdf]

Supplementary Information

S1 Table. Overview literature studies describing brain delivery by cell-penetrating peptides.

CPP	Cargo	Technique	*In vivo/ in vitro*	(Quantitative) result*	Reference
SynB1	Benzylpenicillin	*In situ* brain perfusion in rats (single time) + capillary depletion	*In vivo*	Q: Without SynB1: K_{in} = 0.15 µl/s/g ↔ coupled to SynB1: K_{in} = 1.14 µl/s/g. 80% SynB1 coupled benzylpenicillin in parenchyma.	[1]
SynB1	Dalargin	*In situ* brain perfusion (single time) in rats + antinociceptive test	*In vivo*	Q: Without CPP: V_d = 16.7 µl/g ↔ coupled to SynB1: V_d = 309 µl/g. Enhancement of analgesic activity.	[2]
SynB1	Doxorubicin	*In situ* brain perfusion in rats (single time) + capillary depletion	*In vivo*	Q: Without CPP: K_{in} = 0.25 µl/s/g ↔ coupled to SynB1: K_{in} = 1.50 µl/s/g. 70% SynB1 coupled benzylpenicillin in parenchyma.	[3]
SynB1	Doxorubicin	*In situ* brain perfusion in mice (single time) + capillary depletion	*In vivo*	Q: Without CPP: V_d = < 100 µl/g ↔ coupled to SynB1: V_d = 776.4 µl/g. 60% SynB1 coupled doxorubicin in parenchyma.	[4]
SynB1	PEG-gelatin-siloxane nanoparticles	Determination transcellular transport across co-culture BBB model and determination of PG-GS-SynB particles in brain after IV injection of mice using *in vivo* imaging.	*In vitro/ In vivo*	D: Vectorizing the nanoparticles with the SynB peptide enhances the transport across the BBB *in vitro* as well as *in vivo*.	[18]
SynB3	Dalargin	*In situ* brain perfusion (single time) in rats + antinociceptive test	*In vivo*	Q: Without CPP: V_d = 16.7 µl/g ↔ coupled to SynB3: V_d = 240 µl/g. Enhancement of analgesic activity.	[2]
(L- and D-) SynB3	Doxorubicin	*In situ* brain perfusion in mice (single time) + capillary depletion	*In vivo*	Q: Without CPP: V_d = < 100 µl/g ↔ coupled to (L-en D-) SynB3: V_d = 961.8 (L) and 788.4 (D) µl/g. 50% SynB1 coupled doxorubicin in parenchyma.	[4]
SynB3	Paclitaxel	*In situ* brain perfusion in mice	*In vivo*	D: Vectorized paclitaxel bypasses Pgp at luminal side of BBB.	[5]
SynB3	Morphine-6-glucuronide	*In situ* brain perfusion in mice + antinociceptive tests	*In vivo*	Q: Without CPP: K_{in} = 0.024 µl/s/g ↔ coupled to SynB3: K_{in} = 1.27 µl/s/g. Improvement of pharmacological activity.	[6]

Supplementary Information

S1 Table. Overview literature studies describing brain delivery by cell-penetrating peptides.

CPP	Cargo	Technique	*In vivo/ in vitro*	(Quantitative) result*	Reference
SynB3	Endomorphin-1 (linked by disulfide linkage)	Tail flick antinociceptive test	*In vivo*	Q: Five-fold increase in antinociception compared to EM-1 alone.	[39]
D-Penetratin	Doxorubicin	*In situ* brain perfusion in rats (single time) + capillary depletion.	*In vivo*	Q: Without CPP: K_{in} = 0.25 µl/s/g ↔ coupled to D-Penetratin: K_{in} = 2.14 µl/s/g.<br>70% SynB1 coupled benzylpenicillin in parenchyma.	[3]
Penetratin	-	IV injection → stained with fluorescent tag.	*In vivo*	D: no staining → no brain influx.	[7]
Penetratin	scFvs	Mice were IV injected with scFv-CPP construct. Then the presence of scFv-CPP in brain cryosections was evaluated after fluorescent labeling using a fluorescence microscope.	*In vivo*	D: scFv-CPP construct clearly appeared in brain cells after IV injection.	[26]
Penetratin	Doxorubicin loaded transferrin liposomes	The amount of doxorubicin in brain homogenate was evaluated using HPLC at different time points after IV injection in rats.	*In vivo*	Q: Tf-Penetratin liposomes showed maximal brain penetration after 24h (about 3.67% ID/g).	[27]
Penetratin	PEG-PLA nanoparticles loaded with coumarin-6	*In vivo* imaging and pharmacokinetic and biodistribution studies using LC-MS/MS analysis of coumarin-6 in brain homogenate.	*In vivo*	D: Fluorescence in rat brain was higher for penetratin-NP treated rats than for NP-treated ones. Brain uptake was enhanced when NP was coupled to penetratin.	[28]
Tat 47-57	NEP1-40	Focal ischemia model in rats → evaluate outcome after ischemia + detect presence of vectorized NEP1-40.	*In vivo*	B: Improvement of neurologic outcomes.<br>D: Tat-NEP1-40 detected in brain using Western blot and immunofluorescence.	[8]

Supplementary Information

S1 Table. Overview literature studies describing brain delivery by cell-penetrating peptides.

CPP	Cargo	Technique	*In vivo/ in vitro*	(Quantitative) result*	Reference
Tat 47-57	NR2B9c	Measuring effect of vectorized NR2B9c in a rat stroke model.	*In vivo*	Q: Treatment with Tat-NR2B9c reduced the volume of cerebral infarction with 67% and 87% in cortical infarction volume.	[10]
Tat 47-57	β-galactosidase	Fluorescence confocal microscopy: tissues were dissected from mice 20 min after i.p. injection	*In vivo*	D: - Tat-FITC: strong signals in all areas of brain (not with control FITC).<br>- Tat-β-Gal: brain sections from mice analyzed: 2h post injection, strong activity around capillaries, not in parenchyma, starting from 4h after injection all brain regions showed strong β-Gal activity.<br>- BBB remained intact (Evan's blue albumin complexes not in brain sections).	[11]
Tat 47-57	Green fluorescent protein (GFP)	Evaluation of transcellular transport of Tat-GFP across bEnd-3-astrocyte co-culture layer.	*In vitro*	D: Tat-GFP was able to translocate bEnd-3 cell layer but not astrocyte layer. No influence on barrier integrity observed.	[12]
Tat 47-57	PEG-b-Chol nanoparticles (loaded with ciprofloxacine, quantum dots or FITC)	Confocal microscopy of rat brain sections 4h after IV injection in the tail vein.	*In vivo*	D: Tat-conjugated nanoparticles loaded with FITC or quantum dots crossed the BBB, while FITC and quantum dots alone did not and were localized around blood vessels in the brain.	[13]
Tat 47-57	PEG decorated gelatin-siloxane nanoparticles	*In vivo* imaging (mice) and TEM.	*In vivo*	Q: Tat-modification of the nanoparticles resulted in a quantitatively higher fluorescent signal in the brain than no-Tat nanoparticles (total signal counts of 708.69 ± 4.8 counts/ (sc × s) versus 670.47 ± 8.96 counts/ (sc × s)). TEM analysis revealed that the BBB remained intact.	[14]
Tat 47-57	δ-V1-1 (isozyme specific inhibitor of δ-PKC)	Two *in vivo* models of vascular stress: transient focal ischemia in normotensive rats and chronic hypertension.	*In vivo*	B: δ-V1-1-Tat increased the number of patent microvessels by 92% compared to control (Tat) treated animals and increased cerebral blood flow by 26% following acute focal ischemia (not with Tat peptide alone). In chronic hypertension model, the cerebral blood flow increased by 12%.	[15]

Supplementary Information

S1 Table. Overview literature studies describing brain delivery by cell-penetrating peptides.

**CPP**	**Cargo**	**Technique**	***In vivo/ in vitro***	**(Quantitative) result***	**Reference**
Tat 47-57	PACAP-38	Six hours after i.p. injection of FITC-labeled peptides, the fluorescence intensity of isolated brains was determined.	*In vivo*	B: PACAP-Tat showed a 2.5 fold higher efficiency to traverse the BBB than PACAP.	[16]
Tat 47-57	GDNF	Evaluation of cryosections of mouse brain 4h post i.p. injection using fluorescence microscopy.	*In vivo*	D: The fusion proteins crossed the BBB and transduced the brain parenchyma.	[17]
Tat 47-57	PEG-cholesterol (PEG-b-Col) nanoparticles	Evaluation of presence of FITC loaded PEG-b-Col-Tat nanoparticles in brain cryosections using confocal microscope 4h after IV injection of rats.	*In vivo*	D: PEG-b-Col-Tat particles crossed the BBB.	[19]
Tat 47-57	Bcl-X_L	Evaluation of infarct volume and neurological deficit in ischemic insult mice model.	*In vivo*	D: Tat- Bcl-X_L reduces the infarct volume and neurological deficits when administered before and after ischemic insult.	[22]
Tat 47-57	GDNF	Evaluation whether IV administration of Tat-GDNF prevent brain injury after transient focal ischemia.	*In vivo*	D: After IV administration, the Tat-GDNF protein reaches the ischemic zone and reduced the brain injury and infarction zone.	[23]
Tat 47-57	Ritonavir loaded nanoparticles	Measuring radioactivity of ritonavir in brain tissue digest after decapitation of mice at different time points.	*In vivo*	Q: The brain drug level was 800-fold higher than that with drug in solution at two weeks (0.1 µg/g (solution) versus 80.3 µg/g (Tat-nanoparticles)).	[25]
Tat	Doxorubicin loaded transferrin ligated liposomes	The amount of doxorubicin in brain homogenate was evaluated using HPLC at different time points after IV injection in rats.	*In vivo*	Q: Tf-Penetratin liposomes showed max brain penetration after 24h (about 2.89% ID/g).	[27]

Supplementary Information

S1 Table. Overview literature studies describing brain delivery by cell-penetrating peptides.

CPP	Cargo	Technique	*In vivo/ in vitro*	(Quantitative) result*	Reference
Tat	Quantum dots	Rats were infused intra-arterially and after euthanizing the rat, the brain was isolated and quantum dot fluorescence was evaluated.	*In vivo*	D: If quantum dots are conjugated to Tat, the brain tissue was labeled. Histological data confirm the passage across the endothelial cell line of the blood-brain barrier.	[31]
D-Tat	^{99m}Tc-Tricarbonyl and fluorescein-5-maleimide	Biodistribution was evaluated in mice. At several time points after IV injection, tissues were evaluated by fluorescence microscopy and radiometric analysis.	*In vivo*	D: Little brain permeation was determined.	[32]
Tat	G_3R_6-cholesterol (conjugated to Tat and forms nanoparticles)	4h after i.v. injection of the nanoparticles, rabbit brain sections were evaluated for FITC-loaded nanoparticles using a confocal microscope.	*In vivo*	D: FITC was detected in the brain sections when coupled to CG_3R_6TAT nanoparticles (not if not coupled to nanoparticles), indicating the nanoparticles cross the BBB.	[34]
Tat 47-57	Cholesterol liposomes loaded with coumarin-6	After i.v. injection of the coumarin-6 loaded TAT-liposomes in mice, the coumarin-6 concentration was determined in brain tissue.	*In vivo*	D: The AUC (0-t) for TAT-liposomes was 1.79 to 2.54 times higher than non-conjugated liposomes.	[35]

Supplementary Information
S1 Table. Overview literature studies describing brain delivery by cell-penetrating peptides.

CPP	Cargo	Technique	*In vivo/ in vitro*	(Quantitative) result*	Reference
(D-)Tat 47-57	Liposomes	*In vivo* biodistribution study in mice of (D-)Tat-coupled liposomes loaded with coumarin-6. At different time points after IV injection, the concentration of coumarin-6 was determined *i.a.* in brain tissue. Capillary depletion study was used to demonstrate distribution to brain parenchyma.	*In vivo*	Q: The concentration of coumarin-6 delivered using (D-)Tat-coupled liposomes was 2 to 2.5 times higher than liposomes coupled to a Tat analogue with basic residues replaced by glycine and glutamic acid residues.	[36]
Tat 48-57	FITC doped silica nanoparticles (FSNPs)	The Tat-FSNPs were intra-arterially injected into rats. After completing the procedure, the rats were decapitated and the brain was sliced into 4 pieces and imaged using a fluorescence microscope.	*In vivo*	D: The images confirm labeling of branches of the right middle cerebral artery. Thus, not the nanoparticles crossed the blood-brain barrier.	[37]
Tat 46-57	Doxorubicin loaded nanoparticles (and co-modified with T7 ligand)	*In vivo* imaging and evaluation of survival time of tumor bearing mice.	*In vivo*	D/B: Compared to control liposomes, the TAT-T7 co-modified doxorubicin-loaded liposomes markedly accumulated in the glioma brain tumor. Also the survival time of these mice significantly increased.	[40]
Tat 47-57	VIP	Efficiency assay of traversing the BBB by fluorimetry; food intake assay and evaluation of effect on scopolamine induced amnesia.	*In vivo*	Q: After i.p. injection, the brain uptake efficiency of VIP-TAT (1.81) was twice as high as that of VIP (0.78).<br>B: VIP-TAT had a significantly stronger anorexigenic effect than VIP.<br>Q/B: Administration of VIP-TAT significantly inhibited stronger than VIP alone the reduction of the latent time induced by scopolamine.	[41]

Supplementary Information

S1 Table. Overview literature studies describing brain delivery by cell-penetrating peptides.

CPP	Cargo	Technique	*In vivo/ in vitro*	(Quantitative) result*	Reference
Tat 47-57	BRBP1 (and linked to the proapoptotic peptide KLA as well)	IV injection of fluorescently labeled BRBP1-TAT-KLA in mice with breast cancer brain metastasis.	*In vivo*	D: Compared to TAT-KLA, BRBP1-TAT-KLA showed higher fluorescence intensity in the brain metastasis lesions.	[44]
R7-myristoylated	-	NIR fluorescence imaging after IV injection + immunohistochemical staining.	*In vivo*	D: Presence of fluorescently labeled vector demonstrated.	[9]
rR9	RVG29-cargo (plasmid DNA)	Evaluation of luciferase activity of in several tissues after injection of rR9-RVG29-pGL3 (gene) construct in the tail vein of mice.	*In vivo*	B: At 72h, the luciferase expression was 3-fold higher than for the control group.	[21]
R8	RGD	Translocation across an *in vitro* BBB model AND *in vivo* imaging in C6 glioma bearing mouse model.	*In vitro/ In vivo*	D: R8-RGD reached the brain and accumulated in the glioma foci. Also *in vitro*, R8-RGD crossed the BBB.	[24]
R8	Liposomes	*In vivo* biodistribution study of R8-coupled liposomes loaded with coumarin-6. At different time points after IV injection, the concentration of coumarin-6 was determined *i.a.* in brain tissue. Capillary depletion study was used to demonstrate distribution to brain parenchyma.	*In vivo*	Q: The concentration of coumarin-6 delivered using R8-coupled liposomes was 3.5 times higher than liposomes coupled to a Tat analogue with basic residues replaced by glycine and glutamic acid residues.	[36]
R2-R5	EM-1 analogs	Antinociceptive test	*In vivo*	D: Unless the decreased affinity for the opioid receptor, the vectorized analogs showed potent antinociceptive effect, partly caused by the improved bioavailability.	[29]

Supplementary Information
S1 Table. Overview literature studies describing brain delivery by cell-penetrating peptides.

CPP	Cargo	Technique	*In vivo/ in vitro*	(Quantitative) result*	Reference
Oligo-arginine	Transferrin ligated liposomes	Rat were IV injected with vectorized liposomes and at different time points, brain was isolated and evaluated using NIR imaging.	*In vivo*	D: The brain penetration of Tf-PR-liposomes was 8-fold greater than plain liposomes.	[30]
R11	No cargo, but FITC labeled.	Evaluation of immunoreactivity (using goat anti-FITC antibodies) in mouse brain sections after systemic administration of 11R-FITC.	*In vivo*	D: Strong immunoreactivity was observed in vessels and surrounding cells of cortex, striatum and thalamus, which was not seen when systemically injecting 11E-FITC.	[43]
pVEC	pVEC covalently attached to gHo (glioma homing sequence) = gHoPe2	Evaluation of presence of FAM-labeled gHoPe2, IV injected in mice, in cryosections of the brain.	*In vivo*	D: In intracranial brain tumor model, the gHoPe2-FAM peptides were present in the intracranial tumors, not in healthy brain tissue. Thus the construct crossed the BBB.	[20]
Mastoparan	Doxorubicin loaded transferrin liposomes	The amount of doxorubicin in brain homogenate was evaluated using HPLC at different time points after IV injection in rats.	*In vivo*	D: Accumulation of Tf-Mastoparan liposomes was lower compared to Tf-Tat and Tf-Penetratin liposomes.	[27]
(RXRRBR)2XB	AMO (antisense morpholino oligonucleotides)	IV injection to mice (single time and multiple time injection) and evaluation of presence of fluorescence in different brain areas using fluorescence microscopy.	*In vivo*	D: Fluorescence was widely detected throughout the brain and increased when multiple injections were given.	[33]
PepFect 32 (PepFect 14-Angiopep-2 construct)	pDNA	*In vitro* Transwell experiment. Transport across the bEnd.3 layer was demonstrated by measuring plasmid transfection in U87 cells.	*In vitro*	Q: PepFect 32 showed the most efficient transfection of the luciferase-expressing plasmid in U87 cells.	[38]

Supplementary Information

S1 Table. Overview literature studies describing brain delivery by cell-penetrating peptides.

CPP	Cargo	Technique	*In vivo/ in vitro*	(Quantitative) result*	Reference
LNP	pDNA	Evaluation of *in vitro* transport of LNP-modified pDNA nanoparticles across the BMEC layer.	*In vitro*	Q: P_{app} of LNP-modified pDNA nanoparticles was 92.43 × 10^{-6} cm/s, while if not LNP-modified, the P_{app} was ≤ 65 × 10^{-6} cm/s.	[42]

* Q: Quantitative result; B: biological effect; D: effect described.

References

[1] Rousselle C., Clair P., Temsamani J., Scherrmann J.-M. Improved Brain Delivery of Benzylpenicillin with a Peptide-vector-mediated Strategy. *Journal of Drug Targeting* **10**, 309-315 (2002).

[2] Rousselle C., Clair P., Smirnova M., Kolesnikov Y., Pasternak G.W., Gac-Breton S., Rees A.R., Scherrmann J.-M., Temsamani J. Improved Brain Uptake and Pharmacological Activity of Dalargin Using a Peptide-Vector-Mediated Strategy. *Journal of Pharmacology and Experimental Therapeutics* **306**, 371-376 (2003).

[3] Rousselle C., Clair P., Lefauconnier J.-M., Kaczorek M., Scherrmann J.-M., Temsamani J. New Advances in the Transport of Doxorubicine through the Blood-brain barrier by a Peptide Vector-Mediated Strategy. *MolecularPharmacology* **57**, 679-686 (2000).

[4] Rousselle C., Smirnova M., Clair P., Lefauconnier J.-M., Chavanieu A., Calas B, Scherrmann J.-M., Temsamani J. Enhanced Delivery of Doxorubicin into the Brain via a Peptide-Vector-Mediated Strategy: Saturation Kinetics and Specificity. *Journal of Pharmacology and Experimental Therapeutics* **296**, 124-131 (2001).

[5] Blanc E., Bonnafous C., Merida P., Cisternino S., Clair P., Scherrmann J.-M., Temsamani J. Peptide-vector strategy bypasses P-glycoprotein efflux, and enhances brain transport and solubility of paclitaxel. *Anti-cancer drugs* **15**, 947-954 (2004).

[6] Temsamani J., Bonnafous C., Rousselle C., Fraisse Y., Clair P., Granier L.-A., Rees A.R., Kaczorek M., Scherrmann J.-M. Improved Brain Uptake and Pharmacological Activity Profile of Morphine-6-Glucuronide Using a Peptide Vector-Mediated Strategy. *Journal of Pharmacology and Experimental Therapeutics* **313**, 712-719 (2005).

[7] Bolton S.J., Jones D.N.C., Darker J.G., Eggleston D.S., Hunter A.J., Walsh F.S. Cellular uptake and spread of the cell-permeable peptide penetratin in adult rat brain. *European Journal of Neuroscience* **12**, 2847-2855 (2000).

[8] Wang Q., Gou X., Xiong L., Jin W., Chen S., Hou L., Xu L. Trans-activator of Transcription-mediated Delivery of NEP1-40 Protein into the Brain Has a Neuroprotective Effect against Focal Cerebral Ischemic Injury via Inhibition of Neuronal Apoptosis. *Anesthesiology* **108**, 1071-1080 (2008).

[9] Pham W., Zhao B.-Q., Lo E.H., Medarova Z., Rosen B., Moore A. Crossing the blood-brain barrier: A potential application of myristoylated polyarginine for in vivo neuroimaging. *NeuroImage* **28**, 287-292 (2005).

[10] Aarts M., Liu Y., Liu L., Besshoh S., Arundine M., Gurd J.W., Wang Y.-T., Salter M.W., Tymianski M. Treatment of Ischemic Brain Damage by Perturbing NMDA Receptor-PSD-95 Protein Interactions. *Science* **298**, 846-850 (2002).

[11] Schwarze S.R., Ho A., Vocero-Akbani A., Dowdy S.F. In Vivo Protein Transduction: Delivery of a Biologically Active Protein into the Mouse. *Science* **285**, 1569-1572 (1999).

[12] Simon M.J., Kang W.H., Gao S., Banta S., Morrison III B. TAT Is Not Capable of Transcellular Delivery Across an Intact Endothelial Monolayer *In Vitro*. *Annals of Biomedical Engineering* **39**, 394-401 (2010).

[13] Liu L., Guo K., Lu J., Venkatraman S.S., Lu D., Ng K.C., Ling E.-A., Moochhala S., Yang Y.-Y. Biologically active core/shell nanoparticles self-assembled from cholesterol-terminated PEG-TAT for drug delivery across the blood-brain barrier. *Biomaterials* **29**, 1509-1517 (2008).

[14] Tian X.-h., Wei F., Wang T.-x., Wang D., Wang J., Lin X.-n., Wang P., Ren L. Blood-brain barrier transport of Tat peptide and polyethylene glycol decorated gelatin-siloxane nanoparticle. *Materials Letters* **68**, 94-96 (2012).

[15] Bright R., Steinberg G.K., Mochly-Rosen D. δPKG mediates microcerebrovascular dysfunction in acute ischemia and in chronic hypertensive stress in vivo. *Brain Research* **1144**, 146-155 (2007).

[16] Yu R., Guo X., Huang L., Zeng Z., Zhang H. The novel peptide PACAP-TAT with enhanced traversing ability attenuates the severe injury induced by repeated smoke inhalation. *Peptides* **38**, 142-149 (2012).

[17] Dietz G.P.H., Valbuena P.C., Dietz B., Meuer K., Müller P., Weishaupt J.H., Bähr M. Application of a blood-brain-barrier-penetrating form of GDNF in a mouse model for Parkinson's disease. *Brain Research* **1082**, 61-66 (2006).

[18] Tian X.-h., Wei F., Wang T.-x., Wang P., Lin X.-n., Wang J., Wang D., Ren L. In vitro and in vivo studies on gelatin-siloxane nanoparticles conjugated with SynB peptide to increase drug delivery to the brain. *International Journal of Nanomedicine* **7**, 1031-1041 (2012).

[19] Liu L., Venkatraman S.S., Yang Y.-Y., Guo K., Lu J., He B., Moochhala S., Kan L. Polymeric Micelles Anchored With TAT for Delivery of Antibiotics Across the Blood-Brain Barrier. *Peptide Science* **90**, 617-623 (2008).

[20] Eriste E., Kurrikoff K., Suhorutsenko J., Oskolkov N., Copolovici D.M., Jones S., Laakkonen P., Howl J., Langel Ü. Peptide-Based Glioma-Targeted Drug Delivery Vector gHoPe2. *Bioconjugate Chemistry* **24**, 305-313 (2013).

[21] Gong C., Li X., Xu L., Zhang Y.-H. Target delivery of a gene into the brain using the RVG29-oligoarginine peptide. *Biomaterials* **33**, 3456-3463 (2012).

[22] Kilic E., Dietz G.P.H., Hermann D.M., Bähr M. Intravenous TAT-Bcl-XL Is Protective after Middle Cerebral Artery Occlusion in Mice. *Annals of Neurology* **52**, 617-622 (2002).

[23] Kilic Ü, Kilic E., Dietz G.P.H., Bähr M. Intravenous TAT-GDNF Is Protective After Focal Cerebral Ischemia in Mice. *Stroke* **34**, 1304-1310 (2003).

[24] Liu Y., Ran R., Chen J., Kuang Q., Tang J., Mei L., Zhang Q., Gao H., Zhang Z., He Q. Paclitaxel loaded liposomes decorated with a multifunctional tandem peptide for glioma targeting. *Biomaterials* **35**, 4835-4847 (2014).

[25] Rao K.S., Reddy M.K., Horning J.L., Labhasetwar V. TAT-conjugated nanoparticles for the CNS delivery of anti-HIV drugs. *Biomaterials* **29**, 4429-4438 (2008).

[26] Skrlj N., Drevensek G., Hudoklin S., Romih R., Curin Serbec V., Dolinar M. Recombinant Single-Chain Antibody with the Trojan Peptide Penetratin Positioned in the Linker Region Enables Cargo Transfer Across the Blood-Brain Barrier. *Applied Biochemistry and Biotechnology* **169**, 159-169 (2013).

[27] Sharma G., Modgil A., Zhong T., Sun C., Singh J. Influence of Short-Chain Cell-Penetrating Peptides on Transport of Doxorubicin Encapsulating Receptor-Targeted Liposomes Across Brain-Endothelial Barrier. *Pharmaceutical Research* **31**, 1194-1209 (2014).

[28] Xia H., Gao X., Gu G., Liu Z., Hu Q., Tu Y., Song, Q., Yao L., Pang Z., Jiang X., Chen J., Chen H. Penetratin-functionalized PEG-PLA nanoparticles for brain drug delivery. *International Journal of Pharmaceutics* **436**, 840-850 (2012).

[29] Wang C.-l., Guo C., Wang Y.-q., Zhou Y., Li Q., Ni L.-m., Wang R. Synthesis and antinociceptive effects of endomorphin-1 analogs with C-terminal linked by oligoarginine. *Peptides* **32**, 293-299 (2011).

[30] Sharma G., Modgil A., Layek B., Arora K., Sun C., Law B., Singh J. Cell penetrating peptide tethered bi-ligand liposomes for delivery to brain *in vivo*: Biodistribution and transfection. *Journal of Controlled Release* **167**, 1-10 (2013).

[31] Santra S., Yang H., Stanley J.T., Holloway P.H., Moudgil B.M., Walter G., Mericle R.A. Rapid and effective labeling of brain tissue using TAT-conjugated CdS:Mn/ZnS quantum dots. *Chemical Communications* **25**, 3144-3146 (2005).

[32] Bullock K.E., Dyszlewski M., Prior J.L. Pica C.M., Sharma V., Piwnica-Worms D. Characterization of Novel Histidine-Tagged Tat-Peptide Complexes Dual-Labeled with ^{99m}Tc-Tricarbonyl and Fluorescein for Scintigraphy and Fluorescence Microscopy. *Bioconjugate Chemistry* **13**, 1226-1237 (2002).

[33] Du L, Kayali R, Bertoni C, Fike F, Hu H, et al. (2011) Arginine-rich cell-penetrating peptide dramatically enhances AMO-mediated ATM aberrant splicing correction and enables delivery to brain and cerebellum. Hum Mol Genet 20: 3151-3160.

[34] Wang H, Xu K, Liu L, Tan JPK, Chen Y, et al. (2010) The efficacy of self-assembled cationic antimicrobial peptide nanoparticles against *Cryptococcus neoformans* for the treatment of meningitis. Biomaterials 31: 2874-2881.

[35] Qin Y, Chen H, Yuan W, Kuai R, Zhang Q, et al. (2011) Liposome formulated with TAT-modified cholesterol for enhancing the brain delivery. Int J Pharm 419: 85-95.

[36] Qin Y, Zhang Q, Chen H, Yuan W, Kuai R, et al. (2012) Comparison of four different peptides to enhance accumulation of liposomes into the brain. J Drug Target 20: 235-245.

[37] Santra S, Yang H, Dutta D, Stanley JT, Holloway PH, et al. (2004) TAT conjugated, FITC doped silica nanoparticles for bioimaging applications. Chem Commun 2004: 2810-2811.

[38] Srimanee A, Regberg J, Hallbrink M, Kurrikoff K, Veiman K-L, Vajragupta O, Langel U (2014) Peptide-Based Delivery of Oligonucleotides Across Blood-Brain Barrier Model. Int J Pept Res Ther 20: 169-178.

[39] Liu H, Zhang W, Ma L, Fan L, Gao F, Ni J, Wang R (2014) The improved blood-brain barrier permeability of endomorphin-1 using the cell-penetrating peptide SynB3 with three different linkages. Int J Pharm 476: 1-8.

[40] Zong T, Mei L, Gao H, Cai W, Zhu P, Shi K, Chen J, Wang Y, Gao F, He Q (2014) Synergistic Dual-Ligand Doxorubicin Liposomes Improve Targeting and Therapeutic Efficacy of Brain Glioma in Animals. Mol Pharmaceutics 11: 2346-2357.

[41] Yu R, Yang Y, Cui Z, Zheng L, Zeng Z, Zhang H (2014) Novel peptide VIP-TAT with higher affinity for PAC1 inhibited scopolamine induced amnesia. Peptides 60: 41-50.

[42] Yao H, Wang K, Wang Y, Wang S, Li J, Lou J, Ye L, Yan X, Lu W, Huang R (2015) Enhanced blood-brain barrier penetration and glioma therapy mediated by a new peptide modified gene delivery system. Biomaterials 37: 345-352.

[43] Gotanda Y, Wei F-Y, Haranda H, Ohta K, Nakamura K-i, Tomizawa K, Ushijima K (2014) Efficient Transduction of 11 Poly-arginine Peptide in an Ischemic Lesion of Mouse Brain. J Stroke Cerebrovasc 23: 2023-2030.

[44] Fu B, Long W, Zhang Y, Zhang A, Miao F, Shen Y, Pan N, Gan G, Nie F, He Y, Zhang J, Teng G (2015) Enhanced antitumor effects of the BRBP1 compound peptide BRBP1-TAT-KLA on human brain metastatic breast cancer. Sci Rep 5: 8029.

Remarks:

Ref [7]: Penetratin (directly injected in brain) cause in a dose > 1 µg neurotoxic cell death and recruitment of inflammatory cells. At doses < 1 µg less pronounced toxic effect.
