# Supplementary material for: Cell-Penetrating Peptides Selectively Cross the Blood-Brain Barrier In Vivo
Source: PLoS One. 2015 Oct 14;10(10):e0139652. doi: 10.1371/journal.pone.0139652 (PMC4605843; doi:10.1371/journal.pone.0139652)
Supplement: S1 Text — (PDF) [file pone.0139652.s004.pdf]

S1 Text. Oasis® WCX SPE sample preparation procedure of the serum samples of TP10 and TP10-2.

At the specified time points, 100 µl aliquots were transferred to tubes containing 100 µl of a 4% (V/V) phosphoric acid solution and were consequently heated at 95°C during 5 min. After cooling the samples on an ice-batch, interfering compounds were removed by solid phase extraction using a positive pressure-96 processor (Waters). Before application of the acidified sample on the Oasis® WCX µelution plate, which was conditioned with 200 µl of methanol and equilibrated with 200 µl ultrapure water, the internal standard was added (TP10 for TP10-2 and vice versa). After loading the samples, the Oasis® WCX µelution plate was washed using successively 200 µl of a 5% ammoniumhydroxide and 200 µl of a 70% acetonitrile solution. The peptides were eluted using four times 25 µL of a 1% TFA in 90% acetonitrile solution. The resulting eluate was analyzed by HPLC-UV.
